# Synergies in psychedelic-assisted therapy: a qualitative interview study of psychotherapeutic processes

**DOI:** 10.3389/fpsyt.2026.1771726

**Published:** 2026-04-01

**Authors:** Jonathan Stellmacher, Christopher Schmidt, Helena D. Aicher, Kae Eichel, Eva-Lotta Brakemeier, Uwe Herwig

**Affiliations:** 1 Institut für Psychologie, Universität Greifswald, Greifswald, Germany; 2 MIND Foundation, Berlin, Germany; 3 Department of Psychology, Humboldt-Universität zu Berlin, Berlin, Germany; 4 Clinical Research Center for Substance-Assisted Therapy, Division of Medicine, University of Basel, Basel, Switzerland; 5 Swiss Medical Society for Psychedelic Therapy (SÄPT), Berne, Germany; 6 Department of Psychiatry and Psychotherapy, Center for Psychiatry Reichenau, Reichenau, Germany; 7 Departement of Psychiatry and Psychotherapy III, Universität Ulm, Ulm, Germany; 8 University Hospital of Psychiatry, Universität Zürich, Zürich, Switzerland

**Keywords:** psychedelic-assisted therapy, psychopharmacological effects, psychotherapeutic factors, psychotherapeutic processes, psychotherapeutic setting, psychotherapeutic techniques, thematic analysis, qualitative interviews

## Abstract

Research on the therapeutic effects of psychedelics in psychiatry, commonly referred to as Psychedelic-Assisted Therapy (PAT), has expanded substantially in recent years. The context-dependent nature of psychedelics has sparked discussion about the importance of the psychotherapeutic environment in achieving beneficial outcomes. This study explores the contribution of psychotherapeutic factors on PAT in Switzerland, where psychedelic treatments can be implemented within long-term clinical frameworks. Seven semi-structured interviews were conducted with Swiss therapists to explore how they frame psychedelic treatments and the role of the psychotherapeutic setting in facilitating therapeutic outcomes. Thereby, individual experiences of the patients as reported by the therapists, were particularly considered. Thematic analysis identified two main themes, each with several sub-themes. The first theme revealed that while psychotherapeutic techniques are adapted to PAT, they retain similarities to non-psychedelic psychotherapy practices, supporting patients in having meaningful therapeutic experiences. The second theme describes a synergistic relationship between psychedelics and psychotherapy, amplifying underlying general psychotherapeutic factors such as trust, a sense of profundity, and the emergence of therapeutic experiences. The interviewed therapists agreed that psychedelics work as unspecific catalysts for psychotherapeutic processes, while still acknowledging the potential for psychopharmacological effects or the interaction between psychedelics and psychotherapy to create unique psychotherapeutic processes. Findings from our sample suggest that, for specific indications, incorporating psychedelics into long-term psychotherapeutic treatment may strengthen therapeutic processes. Future research could investigate the efficacy of PAT within the framework of specific psychotherapeutic modalities or in different settings, including prospective quantitative assessments of outcomes. Ultimately, clarifying mechanisms of action of PAT may help to enhance its efficacy and potentially to integrate psychedelic treatments into mainstream mental health care.

## Introduction

Over the past decade, interest in the therapeutic use of psychedelics has seen a resurgence (1–3). Studies have investigated the potential applications of psychedelic-assisted therapy (PAT), focusing on the effects of substances such as psilocybin, lysergic acid diethylamide (LSD), and 3,4-methylenedioxymethamphetamine (MDMA), on various psychiatric disorders. Recent review articles have reported reliable effects of psilocybin-assisted treatments on depression (4) and the impact of MDMA-assisted therapy on post-traumatic stress disorder (PTSD) (5), demonstrating a reduction in psychiatric symptoms. Additionally, studies have investigated the effects of PAT on addiction disorders, anxiety disorders, and end-of-life distress (6). All these studies showed promising results in reducing psychiatric symptoms.

The therapeutic use of psychedelics in psychiatry is being practised in two main domains: study-settings, investigating the efficacy of psychedelics in treating psychiatric disorders, and clinical application in a small number of countries (Australia, Canada, Switzerland) where PAT can be conducted for instance under restricted conditions with permissions outside of study settings (7–9). In either case, PAT sessions are supervised by specifically trained therapists. In this study, the term “therapist” is defined as healthcare professional with a degree in medicine or psychology with additional psychotherapy training.

### The role of psychotherapy in PAT

One of the supposed therapeutic mechanisms of psychedelics, from a neurobiological point of view, is their ability to induce a “window of neuroplasticity” that extends beyond the acute effects (10). This may lead to the modification of neural circuits, promoting changes in behaviour, indicating a possible context-dependent effect of psychedelics. In this case, ‘context’ may refer to environmental factors, such as the room where the psychedelic is taken and whether the person is alone or with others, the individual’s current psychological state and preparation before the treatment session, as well as possible psychotherapeutic integration of the experiences following the session (3). These factors, commonly known as ‘set and setting’, are essential when treating patients with psychedelics, underscoring the importance of integrating PAT within a controlled environment (11). The definition of a “controlled environment” and its significance for PAT has recently sparked discussion. Goodwin et al. (12) argue that this primarily involves passive supervision to ensure patient safety during the acute effects of psychedelics, suggesting that the therapeutic outcomes of PAT should be attributed mainly to the psychopharmacological properties of the substances used. In contrast, Gründer et al. (13) emphasize that due to the context-dependent nature of psychedelics, their therapeutic effects cannot be considered in isolation from the environments in which they are administered, calling for a greater focus on the significance of psychotherapeutic settings. This discussion highlights the need to assess how far the therapeutic results observed in PAT may arise from the psychedelics themselves, the psychotherapeutic context in which they are administered, or an interaction between the two. Addressing this issue may be valuable for future development of psychedelic settings. To date, the level of psychological support provided in PAT studies varies and detailed descriptions of the psychotherapeutic framework are often not primary issues (14). The psychotherapeutic setting can range from basic support, such as a few counselling sessions before and after psychedelic administration with basic supervision during the dosing session where therapists may intervene only in crisis situations, as often applied in research contexts (12, 15), to more intensive approaches in clinical settings where psychedelics are integrated into yearlong, structured psychotherapies (16). Wolff et al. (17) found that people can have psychotherapeutic or psychotherapy-like experiences when using psychedelics in different contexts outside of clinical use or trials, which can be considered within the framework of common factors of psychotherapy (e.g., resource activation, problem actuation, mastery and clarification) (18, 19). The occurrence of therapeutic experiences might be related to different settings and different motives with which people use psychedelics. Further, the creation of a favourable therapeutic context may support the occurrence of therapeutically valuable experiences in dosing sessions and in PAT in general.

### Psychedelic-assisted therapy in research and swiss clinical practise

Ethical considerations preclude the administration of psychedelics without supervision (20, 21), as the consumption of psychedelics in unsupervised settings bares the potential of intensifying symptoms or leading to negative experiences, such as anxiety or intense, unpleasant and psychologically potentially challenging experiences (often referred to colloquially as “bad trips”) (22, 23). Additionally, such settings may increase the likelihood of reckless behaviour, further emphasizing the importance of thorough pre-screening and controlled environments (24). Notably, even challenging experiences during PAT can contribute to therapeutic outcomes when appropriately managed (25). Therefore, recent PAT studies consistently include psychological support delivered by trained therapists. These studies typically standardize the number of preparation and integration sessions, while offering additional sessions to accommodate individual needs (26). Typically, patients participate in one to two dosing sessions throughout the study (27). The therapists conducting the PAT treatment usually only treat the patients for the duration of the study, after which they return to psychopharmacological treatments or outpatient psychotherapists to continue their previously established therapy. Most research so far has focused on investigating PAT in individual settings (6). Furthermore, from a methodological perspective, research on psychedelic-assisted therapy faces challenges regarding placebo conditions and blinding in randomized, double-blind study designs, which necessitates a particularly critical interpretation of efficacy findings (28). These methodological limitations have also played a role in recent regulatory decisions in the United States, including the FDA’s decision to withhold approval of MDMA-assisted therapy for PTSD-treatment (29).

In Switzerland, the treatment of specific patients resistant to standard treatment with psychedelics by healthcare professionals has increasingly been carried out since 2014 within the frame of an exceptional single case permission. For each patient, strict requirements from the Federal Office of Public Health must be met for the treating therapist to receive permission to administer psychedelics. Therapists must submit a PAT application to the Federal Office of Public Health for each patient, with requirements checked individually for each case. Upon approval, permissions for therapists to treat the specific patient are valid for one year, but can be extended if indicated. Therefore, psychedelic sessions may be integrated for long durations into ongoing psychotherapy, allowing for fluid transitions between traditional and psychedelic treatments, with psychotherapeutic material from dosing sessions incorporated into regular psychotherapy. PAT in Switzerland is conducted in both group and individual settings, depending on the preferences of both the therapist and the patient (30).

### Research question

While recent studies assessing psychedelics in psychiatric indications have typically incorporated psychotherapeutic supervision within PAT protocols, they have largely concentrated on evaluating the pharmacological effects of the psychedelic substance itself (31, 32). Although these studies were conducted in a psychotherapeutic context, the specific role and influence of the psychotherapeutic setting have not been comprehensively explored (33, 34), thereby contributing to the ongoing debate between the positions articulated between Goodwin et al. (12) and Gründer et al. (13). This study investigates PAT as it is applied for the treatment of psychiatric disorders, i.e. conditions that are typically addressed within psychotherapeutic frameworks, and focuses on therapists with psychiatric or psychotherapeutic qualifications. We examine how PAT is practised in Switzerland, where psychotherapy usually adopts a central role in treating psychiatric patients, complemented by the occasional administration of psychedelics. Given the ongoing debate surrounding the importance of the psychotherapeutic context of PAT, it remains underexplored how specific psychotherapeutic components might influence treatment outcomes (14, 33). Since interviewing psychotherapists practicing PAT, we assume that psychotherapy plays an important role in psychedelic treatments for psychiatric patients. Rather than asking whether psychotherapy is important in PAT, this study explores how and why psychotherapeutic processes contribute to PAT in psychiatry, and to what extent they interact with the psychedelic experience. Additionally, to investigate these aspects inductively, three different perspectives regarding the association between psychedelics and psychotherapy are explored, representing varying levels of interdependence:

Perspective 1: Psychedelics work through psychopharmacology; psychotherapy is necessary to ensure the safety of this process.

Perspective 2: Psychedelics are unspecific catalysts of specific psychotherapeutic processes.

Perspective 3: Psychedelic-assisted therapy constitutes its own class of psychotherapeutic processes.

Perspective 1 aims to address the assumption that psychedelics exert therapeutic effects primarily through their pharmacological action, with psychotherapy serving as a support mechanism to safeguard the process and manage patient safety, a position consistent with the perspective articulated by Goodwin et al. (12). Perspective 2 postulates the amplification of psychotherapeutic processes by the effects of psychedelics, enhancing or intensifying psychological processes that would naturally arise in traditional therapy, consistent with arguments outlined by Gründer et al. (13). Perspective 3 addresses the possibility that PAT constitutes a distinct class of psychotherapeutic processes, characterized by forms of therapeutic experience that cannot be fully reduced to either pharmacological effects or conventional psychotherapeutic mechanisms. While this perspective is not explicitly articulated within the psychopharmacological–psychotherapeutic debate, it is introduced here to explore the potential of PAT as an emergent therapeutic domain characterized by a high degree of interdependence between psychedelics and psychotherapy. This study aims to deepen the understanding of the role of psychotherapy within PAT.

## Materials and methods

Interviews were conducted with Swiss therapists practising PAT under special licences from the Federal Office of Public Health, outside of research settings. These interviews focused on the therapists’ psychotherapeutic behaviour and attitudes, and how these factors influenced the PAT process according to the therapists’ perspective. The collected data were analysed using a combination of inductive and deductive thematic analysis. While this research did not target any specific diagnosis, the spectrum of conditions typically addressed with this therapy primarily includes depression and trauma-related disorders. This study was preregistered on OSF.io (https://osf.io/69zxg).

### Participants

The focus on Swiss therapists was motivated by three main reasons: (1) The longer history of psychedelic use in psychotherapeutic contexts in Switzerland allowed access to more experienced therapists. (2) Operating in less standardized settings enables Swiss PAT therapists to practice more flexibly, free from strict research protocols. (3) PAT being integrated into the regular treatment of patients offered insights into the long-term interactions between psychedelics and psychotherapy, examining how psychedelics influence the psychotherapeutic process over time. The inclusion criteria for this study were: (1) having a psychotherapeutic background as either a psychiatrist or a psychological psychotherapist, (2) having treated at least three different patients with psychedelics, and (3) having conducted at least ten psychedelic dosing sessions. The sample consisted of *n* = 7 PAT therapists. Four of the therapists were male, and three were female. Five therapists were trained psychiatrists, while two were trained psychological psychotherapists. Five therapists mentioned a background in depth psychology or psychoanalysis, four in somatic therapy, four in systemic therapy, three in trauma therapy, two in cognitive behavioural therapy, one in humanistic psychotherapy, one in gestalt therapy and one in dialectical behaviour therapy (all therapists had several specializations). The participants were between 30 and 65 years old (*M* = 50.3; *SD* = 12.6), had between 5 and 40 years of experience in psychiatry or psychotherapy (*M* = 22.9; *SD* = 11.0), and between 1 and 20 years of experience in PAT (*M* = 7.0; *SD* = 4.4). The number of individual dosing sessions supervised ranged from 6 to 90, and the number of group dosing sessions supervised ranged from 0 to 300. Six participants (*n* = 6) reported that over 80% of their PAT cases were conducted in clinical settings, while one participant (*n* = 1) reported that 60% of their cases occurred in a study setting. Due to the small number of PAT-therapists in Switzerland, providing more specific details could compromise participant anonymity.

### Procedure

Ethics approval for the study was granted by the Universitätsmedizin Greifswald Ethics Committee (Registration No.: BB 065/24). Participants were recruited by the study team’s professional network. Interested therapists received a consent form via email and had the chance to ask questions before signing. Interview appointments were scheduled at convenient locations for the participants, with four interviews conducted in person. The remaining three interviews were conducted online via Zoom due to scheduling conflicts. Before each interview, the study’s aims and content were reviewed, and participants had another opportunity to ask questions. All interviews were audio-recorded for transcription, with a mean duration of 76 minutes (*SD* = 15.9), ranging from 59 to 101 minutes.

### Semi-structured interview

**Figure 1:**
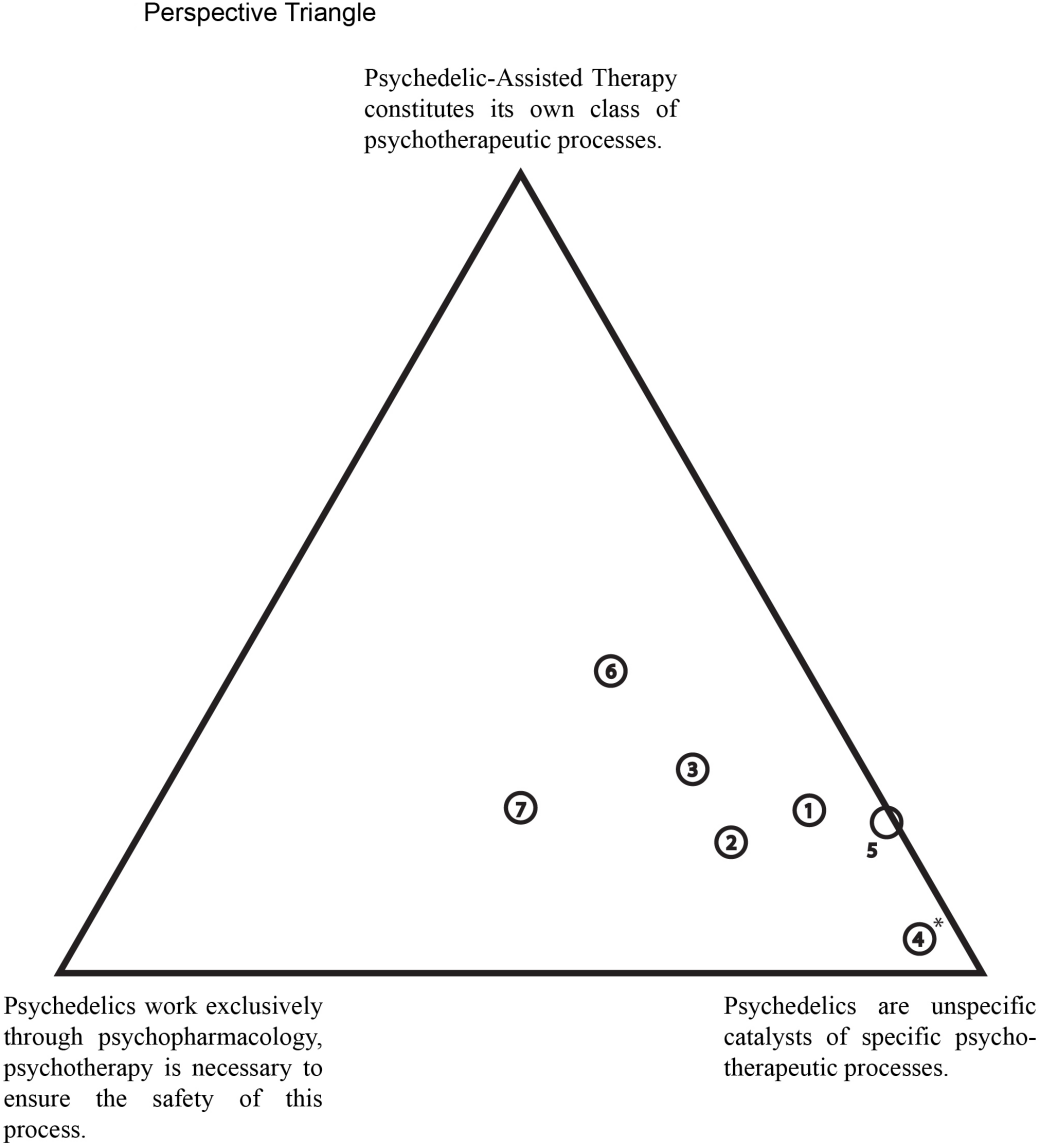
Perspective triangle. At the end of each interview, participants were given this triangular diagram and asked to place a mark indicating their opinion in relation to the perspectives represented at each corner. The numbers inside the circles correspond to therapist’s identifiers, which are consistent with the numbers used in the interview quotes. *Therapist 4 added a note on the print-out: "Psychedelics have specific effects on an individual level".

The semi-structured interview was developed by the author team. The original German interview guide, along with its English translation, can be downloaded as **Supplementary Material**. The interview was divided into three sections: The first section focused on collecting demographic information (age, psychotherapeutic background, experience in psychiatry and PAT). The second section explored the therapist’s approach to PAT, including aspects such as psychotherapeutic stance, the design of the treatment setting, the therapeutic relationship, the therapist’s perceived role, the psychotherapeutic methods employed, and the perceived impact of each aspect on patients. This focus aimed to provide a comprehensive understanding of how therapists shape the PAT context. The third section addressed the perceived interaction between psychedelics and psychotherapy and concluded with showing participants a diagram of an equilateral triangle, with each corner representing a different perspective about the association between psychedelics and psychotherapy. Participants were asked to mark their position based on their agreement with each perspective (**Figure 1**). All interviews were conducted in German. Quotes cited in this study were translated into English using ChatGPT, Version 4, and checked whether they accurately depicted the meaning of the original quotes.

### Analysis

Thematic analysis, as outlined by Terry, Hayfield, Clarke and Braun in the SAGE Handbook of Qualitative Research in Psychology (35), was used to analyse the data. This method involves an iterative process that includes familiarization, coding, theme development, theme review and theme definition. This flexible, interpretive, and exploratory method of data analysis allows for both inductive coding (which accounted for the majority of the data) and deductive coding (used for a smaller number of codes related to the three perspectives). This was favoured because the study addresses a topic that has not been extensively researched.

The audio recordings were transcribed in a two-step process. First, a commercial AI transcription service generated rough verbatim transcripts of the interviews (36). Second, these transcripts were manually reviewed while listening to the recordings to correct errors and remove any personal information that could identify participants. The finalized transcripts were read multiple times to familiarize the researcher with the data.

From the second reading onward, interview segments were coded, assigning semantic and categorical labels using the qualitative data analysis software MAXQDA 24 (37). Codes were refined through an iterative process, candidate themes were identified, thematic maps were constructed, and themes were reviewed and revised until the final thematic map emerged, at which point the themes were clearly defined. All steps of the analysis were conducted by the first author.

Participants were provided with paraphrased versions of their transcripts and questions to clarify any unclear statements, allowing them to comment on the interpretations. Feedback led to clarifications of certain statements, but no major adjustments to data interpretation were required. Additionally, codes used during the analysis were reviewed by a PAT researcher and another researcher unfamiliar with PAT. They were tasked with coding two randomly selected paragraphs from each transcript, and the results were compared and discussed to reach a consensus on interpretations, resulting in further refinements in codes. One notable difference in interpretation involved whether a statement reflected the therapist’s or patient’s perspective, but general content agreement suggests this had limited impact on overall findings.

## Results

**Figure 2:**
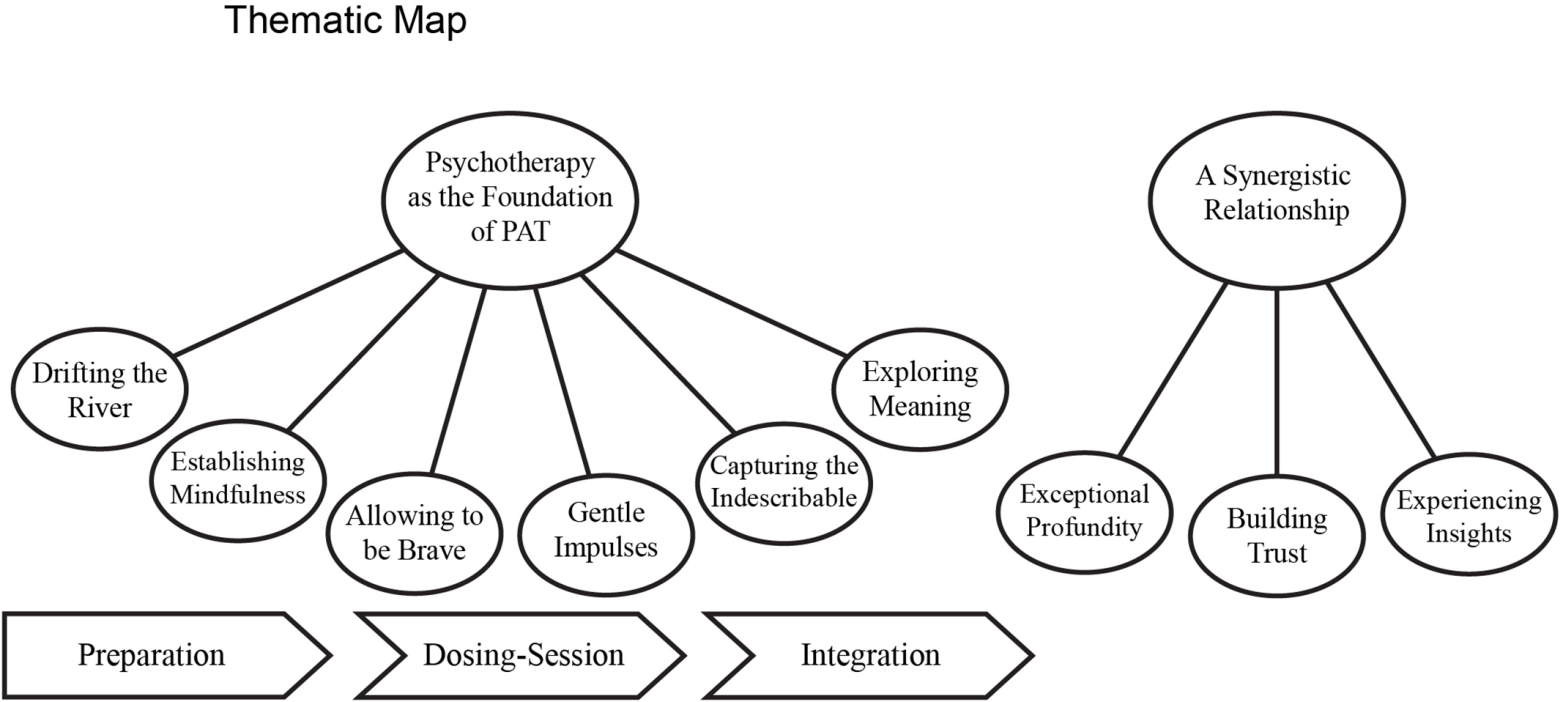
Thematic map. The final thematic map contains two main themes, each containing several sub-themes. Each sub-theme of theme 1 is loosely associated with a PAT-phase.

Note. References such as “D1” refer to additional participant quotes provided in the supplementary document “Additional Thematic Quotes”, which illustrate and support the statements presented in each sub-theme.

Two main themes were derived, each containing several sub-themes: (1) Psychotherapy as the Foundation of PAT and (2) A Synergistic Relationship (**Figure 2**). It is important to note that each participant in this study had their own way of conducting PAT. The following theme definitions and descriptions of PAT phases present a general overview of commonly reported procedures and aspects. However, this does not imply that all therapists included all of these elements or followed a uniform approach. Each aspect mentioned was brought up at least once during the interviews and was considered relevant by the first author. Still, views may differ regarding the importance or implementation of specific elements, and procedures often vary not only between therapists but also from session to session.

### Theme 1: Psychotherapy as the foundation of PAT

This main theme consists of six sub-themes that outline how therapists described their psychotherapeutic work within PAT. While these aspects are also found in traditional psychotherapy, participants reported that they are specifically adapted to the context of PAT. Each sub-theme corresponds to a particular phase of PAT, with two sub-themes associated with each phase. However, this does not mean that sub-themes are exclusive to their phases; psychotherapeutic actions may focus on certain aspects at different stages. For example, mindfulness techniques are primarily taught during preparation, but mindfulness remains important throughout the entire process, such as when therapists guide distressed patients to focus on their breath during dosing sessions or use exercises to reconnect patients with their experiences during integration.

#### Preparation

In the preparation phase of PAT, therapists and patients build rapport and engage in psychoeducation about psychedelics, e.g. discussing potential experiences. This stage helps establish key therapeutic topics and clarifies boundaries. The goal is to enable a sense of safety, trust, and helping patients to engage in the dosing session with a beneficial attitude. Two sub-themes were identified in this phase: (1) Drifting the River and (2) Establishing Mindfulness.

#### Drifting the river

This sub-theme describes how patients’ attitudes are shaped during the psychotherapeutic sessions leading up to the dosing session according to the participants. Patients are encouraged to be self-responsible and curious towards the psychedelic experience, making the decision to actively engage with the upcoming experience and accept it as part of themselves (D1). An open-minded and curious attitude is encouraged for patients (D2). This attitude also involves relinquishing control over one’s emotions to allow deeper processes to emerge and take effect:

*I always say: Imagine you step into a river and just let yourself be carried along. Keep your head above water, but that requires relatively little energy. Just let yourself be carried, whether it’s calm or turbulent, and so on.* (Therapist 2)

Participants reported that in cases where patients are unable to adopt this attitude, complications such as discomfort or anxiety may arise during the psychedelic session (D3). Preventing patients from adopting a resisting attitude can be an important aspect of preparation:

*We don’t want suppressed substance experiences. We don’t want feelings, experiences, or thoughts to be avoided; in fact, one of the goals is to reduce avoidance and promote acceptance.* (Therapist 7)

Participants also discussed other attitudes of patients prior to their psychedelic experiences that might impede a psychedelic session. This included being fixated on PAT as a treatment or on a specific psychedelic substance (D4, D5). Unrealistically high expectations regarding the effectiveness of PAT were frequently mentioned as problematic for the sessions:

*It is important that patients do not feel that they are taking a magic pill and that they can just surrender to the pill and it will fix their entire life.* (Therapist 7)

However, patients tend to learn how to delve deeper into the psychedelic experience as they progress through treatment and gain more experience with psychedelics, being better able to stay open-minded (D6). Overall, cultivating an open mindset characterized by curiosity and realistic expectations may be useful for enabling the therapeutic potential of the psychedelic experience.

##### Establishing mindfulness

This sub-theme emphasizes the value of promoting a mindful attitude for patients, shaping the way patients face the sensations that arise during their psychedelic experience. During the preparation phase, various exercises are practiced to help patients to adopt mindfulness during dosing sessions (M1, M2). Patients are encouraged to remain calm and centred during the dosing session, allowing therapeutic topics to emerge while actively focusing on their emotional resonance with these subjects as they arise:

*The mind is like water, so to speak. When you begin to grasp at something as soon as you see it, the water becomes murkier. [ … ] But if you remain still for as long as possible, with a meditative attitude, you can wait until the water calms on its own, and then you may be able to look down to the bottom. What is there will then reveal itself all the more clearly.* (Therapist 4)

Practising these exercises aims to provide patients with methods to react to potentially emerging emotions such as fear, helping them to have a more comfortable experience (M3). Mindfulness is not only recommended for engaging with inner processes but also for managing external stimuli during the dosing session and is often also proposed in the preparation before sessions:

*With music, it’s not about “whether I like it or not”, but rather about perceiving the resonance within myself. [ … ] It’s about having the willingness to say, “Okay, yes, that’s how I judge”.* (Therapist 3)

Ultimately, cultivating mindfulness equips patients with useful tools to navigate their experiences that may lead to deeper introspection, facilitating therapeutic experiences.

#### Dosing-Session

During this phase of PAT, patients are administered a psychedelic substance and usually experience an altered state of consciousness. The sessions begin with a brief review between therapists and patients to reinforce key points before the substance is administered. Depending on the substance, dosage and individual factors, the major experience can last four to eight hours, after which the effects gradually diminish. Toward the end of the sessions, patients can share their initial reflections with the therapist or peers if the PAT took place in a group setting. Two sub-themes can be designated to this phase: (1) Allowing to be Brave and (2) Gentle Impulses.

##### Allowing to be brave

This sub-theme addresses how the design of the therapy room and the attitudes of therapists during dosing sessions facilitate an environment that encourages patients to relinquish emotional control and engage in self-exploration. The therapeutic setup aims to ensure patient comfort and sense of safety, assisting them to confront challenging emotional topics. To achieve this, the setup includes elements such as spacious rooms, mattresses, blankets, and pillows, along with decorative features like flowers and candles to create a welcoming and supportive atmosphere. Additionally, therapists provide a high-quality sound system for music, snacks, drinks, and options for retreating to quieter areas and ensuring easy restroom access (O1). While not every therapist mentioned all these elements, each aspect was referenced in at least one of the interviews. This pleasant and inviting environment is seen as a condition for fostering a sense of predictability (O2), which supports patients in focusing on their inner experiences and let go of their emotional control:

*I believe we want to create a space where patients can get to know themselves. A place where it should be possible to relinquish a bit of control and engage in the process.* (Therapist 1)

Participants also noted that they viewed their presence and psychotherapeutic attitude as components of the setting.

*And the safe setting is essential. It’s not just about having nice flowers, candles, and a cozy room. It also involves how we, as therapists or as individuals, create an atmosphere where people feel safe.* (Therapist 2)

To cultivate this sense of safety, therapists adopt a nondirective and attentive approach, remaining open and unbiased toward the therapeutic topics that may arise during the session (O3). Some participants mentioned they mirrored the attitude that patients are encouraged to adopt during their sessions in that regard:

*Presence means that I am aware of what is happening within me. Whether I am feeling bored, wishing to do something else, or wondering why nothing is happening here. In other words, the same attitude that the travellers adopt.* (Therapist 3)

If possible, therapists would generally refrain from taking action during dosing sessions, allowing patients to navigate their experiences (O4).

##### Gentle impulses

However, in some cases therapeutic interventions are necessary during the dosing session, as this sub-theme shows. Gentle impulses describe the way therapists respond when patients need support during a psychedelic dosing session. Participants described situations where patients felt unwell, were overwhelmed by emotions, or acted in a way that posed potential risks to themselves or others due to impulsive behaviour following emerging feelings. In such cases, therapists engage with their patients actively. Participants emphasized the need for a careful, steady approach, relying on therapist intuition to determine when and how to intervene, aiming to ensure the interaction remains supportive without being overwhelming (G1). As one therapist noted:

*But I had the feeling she was a bit lost. I can’t say exactly where this impression came from. But I didn’t go right up to her and said, ‘Hey, you’re all alone, let me come over and hug you,’ or anything like that—definitely not. Instead, I just stood up and sat down nearby. She noticed that. Then she turned her head, opened her eyes, and looked at me. Afterward, she told me that it was really important to have someone there to maintain contact with, but she wouldn’t have thought to reach out herself.* (Therapist 5)

Given the altered cognitive states that patients experience during psychedelic sessions, some participants stressed the importance of adapting their interaction style. In practice, active psychotherapeutic interventions rely more on sensory-based approaches—such as touch, sound, music, and even smell in one case—rather than traditional verbal interventions (G2-G5). However, verbal communication can still play a role, being adjusted to altered state of consciousness of patients. For instance, one participant advised to avoid decision-based questions that may overwhelm patients (G6). Therapists also sometimes engage in gentle dialogues that validate and acknowledge patients’ feelings:

*And I just stayed there and validated her in the sense of ‘it’s okay like this.’ Not in order to encourage her more, but more like ‘hey, it’s okay if you do it this way.’ – describing a situation where a patient began screaming and pounding on the floor.* (Therapist 5)

In conclusion, this sub-theme addresses the role of sensory and gentle approaches in facilitating psychotherapeutic interventions during psychedelic dosing sessions. Therapists noted the relevance of attuning to patients’ emotional states and responding with care, allowing for a balanced connection that leads to exploration without overwhelming them.

#### Integration

During this phase of PAT, patients reflect on their experiences from the dosing session, with the goal of integrating the experiences and potential insights into everyday life potentially resulting in positive behavioural adaptation or beneficial shaping of relationships. For most patients, integration begins once the acute effects of the psychedelic subsided on the day of the session and continues in subsequent psychotherapy sessions as well as in everyday life. Therapists’ approaches during this phase were characterized by two sub-themes: (1) Capturing the Indescribable and (2) Exploring Meaning.

##### Capturing the indescribable

Participants emphasized the potential of enabling patients to verbalize their psychedelic experience as a first step in the integration process. This may help patients become aware of emotions and sensations that arose during the session, shedding light on underlying influences that may have affected their lives prior to the experience, often without full cognitive awareness.

*And all of these scenes or situations can later be verbalized with a patient, where the relationship is good or the communication is clear. By doing so, they can be further developed into conscious understanding and used for growth.* (Therapist 4)

*Since he had been able to feel it again, he realized that trust isn’t just a concept, like “just trust me”. [He said] that trust is a real feeling, in a real situation, with a real person.* (Therapist 3)

Verbalization often begins as the effects of the psychedelic start to wane, and cognitive processes begin to normalize (C1). This process can be enriched in a group context, where patients share and reflect on their individual experiences collectively (C2). Some participants encouraged their patients to document their psychedelic experiences by writing a protocol, to facilitate the integration process, which serves as a basis for further exploration (C3). By verbalizing and documenting their experiences, patients may begin to make sense of their psychedelic experience, preparing for deeper integrative work in the subsequent sessions.

##### Exploring meaning

This sub-theme emphasizes the relevance of integrative work that occurs in psychotherapy sessions following psychedelic dosing. Reflecting on these psychedelic experiences is valuable for most patients within the PAT process, as it allows them to explore the meanings behind sensations and insights that arose during their experience. Such exploration may lead to necessary changes that enhance patients’ quality of life (E1). The necessity to explore the deeper meanings of psychedelic experiences varies among patients. Participants noted that some individuals might be capable of drawing their own conclusions without requiring therapeutic intervention. However, for those who struggle with the integration process, therapeutic support is essential (E2). This is particularly the case for patients with severe mental health conditions that frequently undergo PAT.

*And then you’re left with this huge burden [referring to therapeutic material that emerged during a dosing session], feeling worse than before. You know more, you’re aware of the connections again, but it doesn’t get any better at all. And if there’s no therapeutic framework in place, then it’s completely useless. It leads to a deterioration instead.* (Therapist 4)

Moreover, participants highlighted the risk of leaving patients with a sense of incompleteness if the meanings of their experiences are not adequately explored (E3). This was mentioned in cases when patients projected onto their therapist during psychedelic sessions, re-experiencing a past relational dynamic in which the therapist takes on the role of a significant person from the patient’s life. In these cases, the importance of integrative work was emphasized (E4). Such experiences, along with other unpleasant aspects of the psychedelic experience, may require processing to render them therapeutically valuable, thereby providing the opportunity to uncover their underlying significance (E5). Integration sessions also provide patients with the opportunity to give feedback to their therapists. It was added, that psychedelic experiences are unpredictable and it is not always clear what therapeutic actions are necessary, allowing reflection on the interaction is valuable for future dosing sessions. This feedback may even contribute to the psychotherapeutic process on its own (E6). Overall, exploring meaning in the integration phase is beneficial for helping patients process their psychedelic experiences, enabling a sense of understanding and to facilitate change.

### Theme 2: A Synergistic relationship

In contrast to Theme 1, which covers concrete psychotherapeutic actions, Theme 2 addresses the underlying psychotherapeutic processes that may be amplified through the interaction of psychedelics and psychotherapy. Three sub-themes emerged that describe how this interaction potentially intensifies therapeutic dynamics. During the second part of the interviews, many participants referred to psychedelics as “catalysts” for psychotherapeutic processes. The combination of psychotherapy and psychedelics was reported to promote a sense of (1) exceptional profundity in both patients and therapists, (2) building a trusting psychotherapeutic relationship that extends beyond the dosing sessions, and (3) helping patients to gain therapeutic insights and self-awareness, which may be explored further in subsequent psychotherapeutic sessions without psychedelics.

#### Exceptional profundity

This sub-theme centres on the profound depth frequently associated with psychotherapeutic work involving psychedelics, permeating the therapeutic process and amplifying the emotional intensity of patients’ experiences. This profundity was characterized by potentially reducing defence mechanisms, resulting in experiences that may become more intense than those encountered in psychotherapy without psychedelics (P1). This may allow patients to confront challenging topics that are typically avoided (P2). In some instances, this could result in an intense outpouring of emotions (P3). In other cases, this could mean that topics previously addressed in psychotherapy, which were already well understood on a cognitive level, attained a deeper emotional resonance.

*Patients often say, “I’ve been in therapy for ten or twenty years. I know all these phenomena and patterns. I’ve gone over my biography a thousand times. But now, through the psychedelic-assisted therapy, I’ve actually felt or embodied what I previously understood intellectually. I’ve finally experienced what it really means.”* (Therapist 1)

However, this depth was not only associated with patients. Participants articulated that this therapeutic approach may enhance relationships between therapists and patients exceptionally, often surpassing those found in traditional psychotherapy settings.

*I find that the most intense therapeutic relationships I have had in my professional career were during these PAT treatments. It also intensifies the therapeutic relationship.* (Therapist 6)

#### Building trust

Trust was frequently highlighted as being of importance throughout all phases of PAT. Participants emphasized the necessity of establishing a solid foundation of trust between themselves and their patients before initiating any PAT treatment. As one reason for this, participants stated that patients must have confidence in their therapists to enter a psychedelic session with the appropriate mindset (T1). In addition, participants noted that therapists must also trust their patients to remain responsive to therapeutic guidance, even amid intense emotional experiences (T2). Participants described that building trust begins with therapists and patients getting to know each other during the preparation leading up to the dosing session (T3). Some participants noted that they tended to be a bit more transparent with patients about their personal backgrounds compared to psychotherapy without psychedelics, aiming to help patients feel more familiar with the person supervising them during their dosing session (T4). During the dosing session, the intensity of the experience may enhance the level of trust between the therapist and their patients.

*You are having quite a ride during the six hours you spend together. This really brings you together, in a way. Or rather it strengthens the therapeutic relationship quickly.* (Therapist 7)

One therapist proposed that initial trust-building might also be facilitated by using MDMA during the first dosing session. This empathogenic substance might not only lower the risk of a challenging experience but also fosters a deeper connection between patients and therapists (T5).

The absence of a stable therapeutic relationship may lead to significant challenges. One participant recounted a situation in which he struggled to establish a strong bond with a patient seeking PAT, resulting in the patient having a distressing experience while under the influence of LSD.

*I had gotten to know the person during the preparation phase, but I didn’t conduct therapy in the strict sense. [ … ] [He was], I would say today, not accessible to my method. [ … ] After some time, we did an LSD session, and he actually experienced the worst trip, the worst day of his life.* (Therapist 4)

In conclusion, building trust seems to be important to the PAT process, as it forms the foundation for a therapeutic alliance, supporting stability during intense emotional experiences. This trust assists patients in feeling safe when entering the dosing session, potentially promoting a deeper connection throughout treatment, which may strengthen the therapeutic collaboration over time.

#### Experiencing insights

All participants noted that patients could have a diverse range of experiences while under the influence of psychedelics. The combination of the psychotherapeutic setting and the effects of psychedelics often lead to a mental state that allows patients to explore their psyche more freely, unencumbered by their usual thought patterns.

*[ … ] It opens up spaces that are already embedded in our consciousness. It’s not something artificially imposed from the outside, but rather doors that open to realms of awareness that we inherently possess, whether they are past memories or spiritual experiences, etc.* (Therapist 2)

According to several participants, the exact nature of the experiences patients might undergo while under the influence of a psychedelic is unpredictable (X1). However, participants provided a range of examples of the experiences their patients had during dosing sessions. For instance, a patient may develop empathy for themselves or another person during the dosing session, which wasn’t possible before (X2) or may gain sudden, clear insights about their lives:

*It became clear to her that this is one of the reasons she is so overly engaged in everything she does, to avoid being blamed. So no one can accuse her. She had never made this connection so clearly before.* (Therapist 5)

In other examples, participants spoke of patients discovering personal strengths or resources during dosing sessions that they were previously unaware of (X3). They also described patients acknowledging past experiences of suffering—such as childhood trauma—that they had previously downplayed or minimized, now recognizing how difficult those experiences had been at the time (X4). Sometimes the effects of psychedelics can even be so profound, that patients describe them as intense and spiritually meaningful experiences.

*There are also people who simply experience pure bliss and unity, and the dissolution of all opposites. And it is just beautiful.* (Therapist 1)

However, psychedelic experiences are not regularly pleasant. All participants mentioned instances where patients encountered challenging emotions during a dosing session. According to some participants, patients might re-experience situations related to psychological trauma, as the effects of psychedelics can facilitate the emergence of previously avoided or emotionally charged memories (X5) or trigger intense anxiety during a dosing session (X6).

Participants stated that the potential experiences patients might have during their psychedelic session hold the possibility for insight. When these insights are psychotherapeutically harnessed, they can advance the patients’ psychiatric recovery process.

*And to change that, it also requires a new experience, something to be done in the present. Just analysing the past, I believe, is not enough to improve the symptoms. It also takes new experiences for people to realize: ‘Ah, yes, exactly. I am capable. I can do something.’* (Therapist 6)

### Comparison of perspectives regarding the psychotherapeutic impact

When participants were asked open-ended questions about their thoughts on the interaction between psychedelics and psychotherapy, their responses often encompassed multiple perspectives. However, statements aligning with the perspective that posits psychedelics as a distinct class of psychotherapeutic experiences (Perspective 3) were mentioned the least frequently and were accompanied by scepticism (A1). One participant noted that having an unparalleled experience on its own might be special, but it doesn’t necessarily imply that this experience would hold therapeutic value. Such value could emerge through the psychotherapeutic process itself (A2). Another participant suggested that this perspective might hold some truth, though not within the context in which PAT is currently practiced:

*I think if people are sent into a high- or very high-dose range, it may be that certain inclined individuals have experiences and encounters there with, quote-unquote, beings or teachers or insights or principles, which could indeed represent a separate category. But that’s not what’s happening here. [ … ] No, no, this is not its own class. At least not in the way we use it here.* (Therapist 4)

In contrast, statements linking psychedelics to psychopharmacological effects were more prevalent (A3):

*For her, it really is the case that when she takes psilocybin, she experiences significantly fewer depressive symptoms for the next 2 to 3 months. This happens regardless of the content of our sessions or how we approach the therapeutic work. In her case, I genuinely feel that this effect is almost entirely pharmacological.* (Therapist 2)

However, participants who reported a strictly psychopharmacological effect of psychedelics mentioned this in the context of examples they perceived as exceptions. All participants, including those who acknowledged a strictly psychopharmacological effect in some cases, stated that the perceived value of psychedelics lies in their ability to intensify psychotherapeutic processes during psychotherapy sessions (A4).

*They can initiate or accelerate processes like a catalyst or alter the trajectory slightly. This means that one might access certain topics more quickly or intensely, or even trigger a breakthrough. They can serve as catalysts, deepening processes.* (Therapist 1)

Some participants expressed the belief that individuals with sufficient psychological resources might not require therapeutic supervision. However, for the hard-to-treat patients admitted to the PAT program, the access to psychotherapeutic support was seen as essential (A5, A6). Notably, the interplay between psychedelics and psychotherapy was often discussed in the context of neurobiological processes. Many participants highlighted how psychedelics may contribute to the psychotherapeutic process by possibly increasing neuroplasticity, as neurobiological models implicate (A7, A8) (38).

Overall, participants exhibited a preference for the second perspective, which posits that psychedelics act as catalysts for psychotherapeutic processes. This inclination is supported by the frequent mentioning of psychedelics as catalysts during the second part of the interview, which did not focus on exploring these perspectives. This tendency was further reflected in where participants marked their opinions on the triangle representing the three perspectives at the end of the interview (**Figure 1**). All participants positioned their marks close to the lower right corner, which corresponds to the second perspective. Two participants showed mixed opinions, with one equally advocating perspective 1 and 2, and the other equally advocating perspective 2 and 3. However, no participant dismissed perspective 1 and 3 entirely (Therapist 4 did so in the diagram, but not when they spoke about the perspectives). Thus, no participants completely ruled out the possibility of psychopharmacological factors or the potential for psychedelics and psychotherapy to elicit specific processes.

## Discussion

In recent years, the importance of situating PAT within a psychotherapeutic context has been discussed in psychedelic research (12, 13), while current studies rarely emphasize the role of psychotherapy in PAT (14). This study examined psychotherapeutic aspects of PAT in Switzerland, where it is applied in both outpatient and inpatient clinical settings with a strong emphasis on psychotherapy. Treatments are embedded in a long-term psychotherapeutic setting (16). Contents of the individual experiences throughout the dosing sessions are regularly addressed during the continuing psychotherapy. It provides a range of examples that highlight how the PAT process can be actively shaped within a psychotherapeutic context and how this approach complements psychotherapeutic work. During preparation, patients are encouraged to adopt an open and accepting attitude, conducive to therapeutic experiences, also as a strategy to manage challenging emotions. The psychotherapeutic setting is carefully designed to ensure safety, with therapists maintaining a supportive and attentive presence throughout the dosing session, using professional and tailored interactions to accompany patients through their experiences. Sensory-based stimuli, such as touch and sound, are used to elicit therapeutic effects. Post-session, therapists support patients processing their experiences, attempting to enable a sense of meaning regardless of whether the experience was pleasant or unpleasant.

Participants frequently described psychedelics as “catalysts” for psychotherapeutic processes, emphasizing a synergy between psychedelics and psychotherapy that extends beyond the dosing session and enhances the overall psychotherapeutic process. Among other aspects, this synergy appears to cultivate a deeper level of trust between therapists and patients. While building trust is actively focused at the beginning of PAT, it seems to be further deepened through the shared experience of often profound processes during the sessions. These experiences provide the possibility for psychotherapeutic exploration, potentially helping patients to break free from entrenched behavioural patterns and promoting change. This perception of PAT was also present when participants were asked to express their assumption on the association between psychedelics and psychotherapy, as **Figure 1** shows.

Each sub-theme identified in this study has a specific focus on how the interplay between psychedelics and psychotherapy is shaped. However, the themes described in this study are not independent from each other. The mutual influence between the concrete behaviour therapists use to facilitate psychedelic experiences (Theme 1) and the underlying aspects of psychotherapy in PAT (Theme 2) is particularly significant. For instance, trust is an important element throughout the PAT process and can be seen as being part of the mindset that is shaped during preparation and pivotal for therapist-patient interaction during the dosing session. Through mutually experiencing profound emotional processes, the psychotherapeutic relationship may become more trusting, making trust not only a prerequisite for PAT but also a consequence thereof. The profundity of psychedelic experiences may not only be enabled by the substance but also by thorough preparation and the therapist’s attitude, such as remaining attentive and open for gentle interactions when patients are in need. These elements may support deeper introspection, potentially leading to personal insights that can be integrated into ongoing psychotherapy, with recent research confirming the lasting effects of psychedelics (1).

The therapists’ reports suggest that integrating psychedelic treatments into comprehensive psychotherapeutic setting may enhance the therapeutic impact of psychedelics. Participants in this study indicated that psychedelics might act as catalysts within psychotherapy, a view that aligns with early psychedelic research from the 1950s (39) and the psycholytic therapy approach developed in the same period (40). While Aicher et al. (41) have highlighted that many insights now discussed in psychedelic research were already explored in earlier studies, modern literature still lacks robust investigation into the long-term use of psychedelic dosing sessions within a sustained psychotherapeutic framework. At a neurobiological level, research has shown that psychedelics can increase neuroplasticity beyond their psychoactive effects (42), suggesting that structured psychotherapeutic contexts may further promote beneficial change during this heightened state (43). The results of this qualitative study also essentially correspond to the statements made by Gründer et al. (13) that PAT is a form of psychotherapy.

However, this should not imply that PAT must generally be conducted within a comprehensive psychotherapeutic context. While participants principally emphasized the value of combining psychedelics with psychotherapy, some noted instances where a positive change might occur in non-psychotherapeutic settings. A recent study did not find a relation between treatment outcome of psilocybin in depression and hours of psychotherapeutic support (44), however, the range was between 4 and 18 hours in very varying settings with only one or two dosing sessions, such not representing the clinical setting as explored in the context of this study. Although safety considerations necessitate that psychedelics are administered in controlled environments (45), studies that use passive supervision during dosing sessions, with minimal preparation and integration, have also shown beneficial therapeutic outcomes (46). In these cases, consisting of patients with a potentially life-threatening cancer diagnosis and anxiety and/or mood symptoms, the impact of the psychedelic compound, including setting and basic support, were sufficient for a positive outcome. Adding specific psychotherapeutic strategies hypothetically may further augment therapeutic efficacy. Research could also benefit from comparing various approaches to PAT, with differing levels of psychological support, to better understand the influence of setting on therapeutic outcomes.

The participants’ tendency to view psychedelics as catalysts of psychotherapy, emphasizing the role of the psychotherapeutic context, may reflect their professional backgrounds. Perceptions of PAT vary across professions; for example, psychologists, psychiatrists, and social workers have differing views on its benefits and limitations (47). Although these findings are not directly transferable to this study’s participants, who consisted of psychologists and psychiatrists, Armstrong et al. (47) highlights a general lack of consensus on psychedelic effects. This divergence is not new; as described by Hartogsohn (48), distinct psychedelic modalities emerged during the first wave of research, including (1) the Psychotomimetic Modality (aligning with this study’s perspective 1), (2) the Psychotherapeutic Modality (perspective 2), and (3) the Spiritual Modality (perspective 3 from a specific point of view), each associated with specific theories and practices. Participants in this study did not exclude perspective 1 and 3 and even offered cases where these might apply over perspective 2, implying that each perspective may hold validity in its respective domain, depending on the approach and individuals involved. PAT as practiced in this study aligns with perspective 2, but this does not preclude future development of therapeutic applications that emphasize psychedelics’ psychopharmacological effects (49, 50) or the existential and spiritual dimensions and interpretations sometimes occurring in PAT sessions (51).

Certain principles by which PAT is described by the participants in this study (e.g., “drifting the river”) may resemble reports of non-clinical psychedelic practices, such as those sometimes facilitated by trip sitters, guides, or shamans, suggesting that features of the approaches described here may be more specific to psychedelic practice than consistent with conventional psychotherapeutic processes (52, 53). This similarity extends beyond the structural elements of preparation, dosing, and integration to include, for example, relational stances such as maintaining a predominantly non-directive presence during dosing sessions, with gentle verbal or physical interventions only when the individual undergoing the psychedelic experience is in evident need of support (52). Importantly, this overlap may indicate that several processes identified in this study are not specific to distinct psychotherapeutic techniques, but rather reflect transdiagnostic and transdisciplinary relational and experiential factors that become particularly salient in psychedelic-assisted contexts, thereby partially exceeding experiences of conventional psychotherapy. While these perspectives fall outside the scope of our clinical focus, they suggest possible future research avenues to compare therapeutic outcomes across contexts.

Across contemporary psychedelic-assisted therapy studies, elements of cognitive-behavioural therapy are commonly incorporated into therapeutic protocols (54, 55), most prominently the Yale Manual (56). Some authors have suggested that CBT-based approaches may be particularly suitable for PAT due to their emphasis on culturally neutral frameworks, their minimal reliance on speculative models of mind and reality and their extensive empirical evidence base (57). In contrast, the participants of the present study, Swiss psychotherapists representing a broad range of therapeutic orientations with only two reporting a background in CBT, suggest a different interpretation. The findings indicate that the therapeutic value of combining psychotherapy and psychedelics lies less in school-specific techniques than in their interaction with psychotherapeutic characteristics common across approaches, such as a trusting therapeutic relationship or experiences of increased depth and meaningfulness, with several participants additionally highlighting the relevance of body-focused methods. Overall, these results support the view that psychedelics may deepen psychotherapeutic processes largely independently of the underlying therapeutic orientation, exerting their effects within the patient-therapist relationship and the experiential depth of therapeutic change.

According to participants’ reports, PAT can be characterized as a therapeutic approach that allows patients to connect deeply with sensations, emotions, and explore meaning in life. These aspects have been discussed in prior research (58) and are implemented in the preparation of PAT-patients (56, 59). Future studies could focus on these emotional dimensions—such as trust, emotional depth, and meaningful experiences—and investigate how they may impact long-term psychotherapy outcomes. Psychedelics may offer a more direct pathway to engaging patients emotionally, not only during the dosing session but also in the integration phase, where these experiences can be further processed through discussion, interpretation, and reflection with the therapist.

Also, the repeatedly reported observation of emerging, potentially repressed or avoided memories may yield valuable material for psychotherapeutic work. Of course, one should remain aware of the potential risk of false memories (60), however, a thorough evaluation may be warranted.

Innovation in PAT could explore new environments beyond the traditional setup where patients lie down with eyes closed. One participant proposed that mindful walks in nature during dosing could offer therapeutic benefits, potentially enhancing sensory engagement while preserving the core principles of set and setting. Reports of psychedelic use outside therapeutic settings include examples where contact with nature has induced peak experiences, such as profound feelings of universal love and spirituality (61). Gandy et al. (62) also suggested potential mental-health benefits by combining psychedelics with natural settings. Such approaches would require careful planning to avoid overstimulation, but could provide a new dimension to PAT.

Emotional depth is a core aspect of PAT. Current methods are non-directive, aiming to create a supportive environment for introspection (56). Therapists in this study described to use *gentle impulses*, tailoring sensory-based interventions like touch or sound to the patient’s mental state during dosing sessions, encouraging their mindful engagement with their emotional resonance to stimuli. The importance of sensory stimuli is also highlighted by O’Callaghan et al. (63), who emphasize music’s role in fostering emotional resonance. One participant of this study reported that even smell played a role in facilitating a meaningful therapeutic moment, indicating that future developments of PAT could explore a broader range of sensory stimuli to enrich the therapeutic process for patients under the influence of psychedelics.

While experience enriching stimuli, such as music and ambient sounds, are well-established in PAT, therapists shared various instances where physical touch—such as holding a patient’s head, allowing them to lean on a shoulder, or placing a hand on the solar plexus—provided significant comfort and support (64, 65). Several therapists in this study, particularly those with backgrounds in body psychotherapy, recounted experiences where gentle, supportive touch helped to alleviate acute distress or facilitated emotional breakthroughs, aligning with established methods in body-focused therapies (66). However, the use of touch in PAT introduces important ethical considerations, especially regarding consent, particularly in light of the intensified therapeutic relationship also described by participants in this study. Though patients may initially agree to physical contact during preparation, the altered mental states induced by psychedelics can affect a patient’s comfort level (67). A patient who consents to touch beforehand might feel uncomfortable during the session, while another who previously avoided touch might seek it if they experience distress during dosing (68). Given the heightened vulnerability of patients in these altered states, any misuse of physical contact can cause significant harm, as evidenced by past incidents (69, 70). To maintain patients’ safety and integrity, therapists must remain vigilant throughout the session. While recent discussions have started to address the ethical implications of touch in altered states (65, 71), more research is necessary to establish clear guidelines on when and how touch may be safely and ethically incorporated into psychedelic sessions.

This discussion is also important regarding the psychotherapeutic alliance. Several participants noted that the relationship they had with PAT patients was exceptionally deep, personal and trusting. While a strengthened psychotherapeutic alliance may be desirable, given its role as a potential mediator for therapeutic change in both general psychotherapy (72) and PAT specifically (34), such a deepened relationship might present challenges around maintaining boundaries (73). Principally, the role of general psychotherapeutic modes of action has to be discussed in this context (74). This highlights the need for further exploration into the nature of psychotherapeutic relationships in PAT, as the unique depth of the therapist-patient bond in this context may require special attention in PAT guidelines. Phelps (75) has taken an exemplary step in this direction in describing important characteristics of PAT-therapist, including Therapist Self-Awareness and Ethical Integrity.

## Limitations and future studies

Several limitations are associated with this qualitative study. The sample included overall only seven therapists practicing PAT in Switzerland, all recruited from a similar professional background. The shared training background may have led to aligned perspectives, especially since some participants were professionally connected. Moreover, this study did not include non-psychotherapeutic participants which may have further emphasized the importance of a psychotherapeutic context, potentially overlooking perspectives present in non-clinical psychedelic settings that employ similar principles of preparation, support during dosing, and post-session integration, as well as viewpoints that place greater emphasis on the psychopharmacological effects of psychedelics. Although five of the therapists had a medical background, all identified primarily as psychotherapists.

While some countries are currently exploring alternative access models, in many regulatory contexts further Phase 3 clinical trials will be required to establish effectiveness before PAT can be integrated into routine clinical practice. Additionally, rather than focusing on school-specific interventions, future research may profit from integrating established findings from psychotherapy research on common and transdiagnostic factors, which may be particularly relevant in the context of psychedelic-assisted treatment. For instance, processes emphasized in emotion-focused and body-oriented approaches have been shown to enhance the patient’s sense of meaning and strengthen the psychotherapeutic bond. Finally, the specific constraints in the design of PAT-studies regarding placebo-controlled conditions must be accounted (28). Here, however, we focused on reports from clinical experiences, not framed by a study design.

Observational studies following PAT treatments outside of controlled research settings could enhance the findings presented here. Additionally, future research could investigate the potential of regular, structured psychedelic dosing sessions integrated into long-term psychotherapy, examining how factors such as trust, emotional depth, and neuroplasticity influence treatment outcomes. To further explore the role of the psychotherapeutic environment, this approach could be compared to PAT models that involve more passive supervision, as well as those incorporating more directive therapist interventions during dosing sessions.

## Conclusion

The effects of psychedelics seem to be context-dependent (76, 77), and this study highlights how psychotherapy might shape the psychedelic experience in PAT, implying the possibility of a synergistic interaction between the two. We present distinct, concrete psychological themes to offer a more profound understanding of how psychotherapy exerts its effects within PAT on a psychological level. Particularly, we considered the individual patient experiences as reported by the therapists in order to elucidate potential modes of therapeutic effect. While beneficial psychedelic experiences may occur outside of therapeutic settings, the current practice of PAT appears to increase the likelihood of therapeutic experiences and outcomes during dosing sessions, indicating the value of a psychotherapeutic framework. Our results indicate that a key feature of PAT may lie in enhancing the therapeutic relationship and deepening the experiential intensity of the psychotherapeutic process. Given the high variability and inherent difficulty of predicting the course of psychedelic experiences, the presence of a professional therapeutic context for psychiatric patients is strongly recommended, not only for safety reasons but also to optimize therapeutic effects. Though the risk of overwhelming or distressing experiences cannot be fully avoided, even in controlled environments, such experiences may hold therapeutic value if properly integrated in a psychotherapeutic setting. Immediate access to psychological support is recommended to help patients navigate these challenges constructively. The findings of this study suggest that establishing a therapeutic safety net is an ethical requirement for psychedelic treatments in psychiatry, with factors like a strong therapeutic relationship and a safe environment being especially important in the PAT context. Ultimately, the findings of this qualitative study highlight the need of further addressing characteristics of the psychotherapeutic impact in the frame of psychedelic-assisted therapy.

## Data Availability

The datasets for this article are not publicly available due the sensitive nature of the qualitative interview data and data protection regulations under which the study was conducted. Requests to access the datasets should be directed to the corresponding author.

## References

[1] Aday JS Mitzkovitz CM Bloesch EK Davoli CC Davis AK . Long-term effects of psychedelic drugs: A systematic review. Neurosci Biobehav Rev. (2020) 113:179–89. doi: 10.1016/j.neubiorev.2020.03.017, PMID: 32194129

[2] Fonzo GA Nemeroff CB Kalin NH . Psychedelics in psychiatry: oh, what A trip! Am J Psychiatry. (2025) 182:1–5. doi: 10.1176/appi.ajp.20241025, PMID: 39741442

[3] Reiff CM Richman EE Nemeroff CB Carpenter LL Widge AS Rodriguez CI . Psychedelics and psychedelic-assisted psychotherapy. Am J Psychiatry. (2020) 177:391–410. doi: 10.1176/appi.ajp.2019.19010035, PMID: 32098487

[4] Metaxa A.−M. Clarke M . Efficacy of psilocybin for treating symptoms of depression: Systematic review and meta-analysis. BMJ (Clinical Res Ed.). (2024) 385:e078084. doi: 10.1136/bmj-2023-078084, PMID: 38692686 PMC11062320

[5] Shahrour G Sohail K Elrais S Khan MH Javeid J Samdani K . Mdma-assisted psychotherapy for the treatment of PTSD: A systematic review and meta-analysis of randomized controlled trials (RCTs). Neuropsychopharmacol Rep. (2024) 44:672–81. doi: 10.1002/npr2.12485, PMID: 39381877 PMC11609750

[6] Luoma JB Chwyl C Bathje GJ Davis AK Lancelotta R . A meta-analysis of placebo-controlled trials of psychedelic-assisted therapy. J Psychoactive Drugs. (2020) 52:289–99. doi: 10.1080/02791072.2020.1769878, PMID: 32529966 PMC7736164

[7] Aicher HD Müller F Gasser P . Further education in psychedelic-assisted therapy - experiences from Switzerland. BMC Med Educ. (2025) 25:341. doi: 10.1186/s12909-025-06871-y, PMID: 40045361 PMC11881254

[8] Hatfield SP Thornton NLR Greenstien K Glozier N . A taxonomy of regulatory and policy matters relevant to psychedelic-assisted therapy in Australia. Aust New Z J Psychiatry. (2024) 58:571–90. doi: 10.1177/00048674241240597, PMID: 38628079 PMC11193325

[9] Mocanu V Mackay L Christie D Argento E . Safety considerations in the evolving legal landscape of psychedelic-assisted psychotherapy. Subst Abuse Treatment Prevention Policy. (2022) 17:37. doi: 10.1186/s13011-022-00468-0, PMID: 35568884 PMC9107659

[10] Calder AE Hasler G . Towards an understanding of psychedelic-induced neuroplasticity. Neuropsychopharmacology: Off Publ Am Coll Neuropsychopharmacol. (2023) 48:104–12. doi: 10.1038/s41386-022-01389-z, PMID: 36123427 PMC9700802

[11] Hartogsohn I . Set and setting for psychedelic harm reduction In: Current Topics in Behavioral Neurosciences. Berlin, Heidelberg: Springer. (2024). pp. 1–14. doi: 10.1007/7854_2024_509, PMID: 39080241

[12] Goodwin GM Malievskaia E Fonzo GA Nemeroff CB . Must psilocybin always “Assist psychotherapy”? Am J Psychiatry. (2024) 181:20–5. doi: 10.1176/appi.ajp.20221043, PMID: 37434509

[13] Gründer G Brand M Mertens LJ Jungaberle H Kärtner L Scharf DJ . Treatment with psychedelics is psychotherapy: Beyond reductionism. Lancet Psychiatry. (2023) 11:231–6. doi: 10.1016/S2215-0366(23)00363-2, PMID: 38101439

[14] Brennan W Kelman AR Belser AB . A systematic review of reporting practices in psychedelic clinical trials: psychological support, therapy, and psychosocial interventions. Psychedelic Med. (2023) 1:218–29. doi: 10.1089/psymed.2023.0007, PMID: 40046864 PMC11658666

[15] Barber GS Aaronson ST . The emerging field of psychedelic psychotherapy. Curr Psychiatry Rep. (2022) 24:583–90. doi: 10.1007/s11920-022-01363-y, PMID: 36129571 PMC9553847

[16] Aicher HD Gasser P . Treatment recommendations for psychedelic-assisted therapy. Swiss Arch Neurology Psychiatry Psychother. (2024) 175:103–6. doi: 10.4414/sanp.2024.1488043038, PMID: 40251714

[17] Wolff M Evens R Mertens LJ Schmidt C Beck J Rutrecht H . Measuring psychotherapeutic processes in the context of psychedelic experiences: Validation of the General Change Mechanisms Questionnaire (GCMQ). J Psychopharmacol (Oxford England). (2024) 38:432–57. doi: 10.1177/02698811241249698, PMID: 38742761 PMC11102652

[18] Grawe K . Research-informed psychotherapy. Psychother Res. (1997) 7:1–19. doi: 10.1080/10503309712331331843, PMID: 9407471 PMC3330485

[19] Wampold BE Imel ZE . *The great psychotherapy debate: The evidence for what makes psychotherapy work* (Second edition). New York: Routledge Taylor & Francis (2015). doi: 10.4324/9780203582015, PMID:

[20] Alpert MD O’Donnell KC Paleos CA Sola E Stauffer CS Wagner AC . Psychotherapy in psychedelic treatment: safe, evidence-based, and necessary. Am J Psychiatry. (2024) 181:76–7. doi: 10.1176/appi.ajp.20230665, PMID: 38161307

[21] Earleywine M de Leo J Bhayana D Rajanna B Scott K . Psilocybin without psychotherapy: A cart without a horse? Am J Psychiatry. (2024) 181:78–9. doi: 10.1176/appi.ajp.20230572, PMID: 38161299

[22] Branchi I . The double edged sword of neural plasticity: Increasing serotonin levels leads to both greater vulnerability to depression and improved capacity to recover. Psychoneuroendocrinology. (2011) 36:339–51. doi: 10.1016/j.psyneuen.2010.08.011, PMID: 20875703

[23] Ona G . Inside bad trips: Exploring extra-pharmacological factors. J Psychedelic Stud. (2018) 2:53–60. doi: 10.1556/2054.2018.001, PMID: 29951292

[24] Johnson MW Richards WA Griffiths RR . Human hallucinogen research: Guidelines for safety. J Psychopharmacol (Oxford England). (2008) 22:603–20. doi: 10.1177/0269881108093587, PMID: 18593734 PMC3056407

[25] Wolff M Evens R Mertens LJ Koslowski M Betzler F Gründer G . Learning to let go: A cognitive-behavioral model of how psychedelic therapy promotes acceptance. Front Psychiatry. (2020) 11:5. doi: 10.3389/fpsyt.2020.00005, PMID: 32153433 PMC7046795

[26] Mertens LJ Koslowski M Betzler F Evens R Gilles M Jungaberle A . Methodological challenges in psychedelic drug trials: Efficacy and safety of psilocybin in treatment-resistant major depression (EPIsoDE) - Rationale and study design. Neurosci Appl. (2022) 1:100104. doi: 10.1016/j.nsa.2022.100104, PMID: 40656230 PMC12244097

[27] Dawood Hristova JJ Pérez-Jover V . Psychotherapy with psilocybin for depression: systematic review. Behav Sci (Basel Switzerland). (2023) 13:297. doi: 10.3390/bs13040297, PMID: 37102811 PMC10135952

[28] Hieronymus F López E Werin Sjögren H Lundberg J . Control group outcomes in trials of psilocybin, SSRIs, or esketamine for depression: A meta-analysis. JAMA Network Open. (2025) 8:e2524119. doi: 10.1001/jamanetworkopen.2025.24119, PMID: 40736734 PMC12311713

[29] FDA Center for Drug Evaluation and Research . NDA 215455: COMPLETE RESPONSE. Silver Spring, MD, USA: United States Food and Drug Administration (FDA (2024). Available online at: https://download.open.fda.gov/crl/CRL_NDA215455_20240808.pdf (Accessed January 1, 2026).

[30] Liechti ME Gasser P Aicher HD Müller F Hawrot T Schmid Y . Implementing psychedelic-assisted therapy: History and characteristics of the Swiss limited medical use program. Neurosci Appl. (2025) 4:105525. doi: 10.1016/j.nsa.2025.105525, PMID: 40800003 PMC12341733

[31] Carhart-Harris RL Bolstridge M Day CMJ Rucker J Watts R Erritzoe DE . Psilocybin with psychological support for treatment-resistant depression: Six-month follow-up. Psychopharmacology. (2018) 235:399–408. doi: 10.1007/s00213-017-4771-x, PMID: 29119217 PMC5813086

[32] Goodwin GM Aaronson ST Alvarez O Arden PC Baker A Bennett JC . Single-dose psilocybin for a treatment-resistant episode of major depression. New Engl J Med. (2022) 387:1637–48. doi: 10.1056/NEJMoa2206443, PMID: 36322843

[33] Aday JS Horton D Fernandes-Osterhold G O’Donovan A Bradley ER Rosen RC . Psychedelic-assisted psychotherapy: Where is the psychotherapy research? Psychopharmacology. (2024) 241:1517–26. doi: 10.1007/s00213-024-06620-x, PMID: 38782821

[34] Murphy R Kettner H Zeifman R Giribaldi B Kärtner L Martell J . Therapeutic alliance and rapport modulate responses to psilocybin assisted therapy for depression. Front Pharmacol. (2021) 12:788155. doi: 10.3389/fphar.2021.788155, PMID: 35431912 PMC9009076

[35] Willig C Stainton-Rogers W . *The SAGE handbook of qualitative research in psychology* (Second edition). Los Angeles; London; New Delhi; Singapore: Sage reference (2017).

[36] dr. dresing & pehl GmbH . audiotranskription.de: Transkribieren, Codieren & Analysieren mit f4 audiotranskription (2020). Available online at: https://www.audiotranskription.de/ (Accessed November 3, 2024).

[37] VERBI GmbH . *MAXQDA* (Version MAXQDA 24) (2023). Available online at: maxqda.com (Accessed November 3, 2024).

[38] Vargas MV Dunlap LE Dong C Carter SJ Tombari RJ Jami SA . Psychedelics promote neuroplasticity through the activation of intracellular 5-HT2A receptors. Sci (New York N.Y.). (2023) 379:700–6. doi: 10.1126/science.adf0435, PMID: 36795823 PMC10108900

[39] Eisner BG Cohen S . Psychotherapy with lysergic acid diethylamide. J Nervous Ment Dis. (1958) 127:528–39. doi: 10.1097/00005053-195812000-00006, PMID: 13621221

[40] Passie T Guss J Krähenmann R . Lower-dose psycholytic therapy - A neglected approach. Front Psychiatry. (2022) 13:1020505. doi: 10.3389/fpsyt.2022.1020505, PMID: 36532196 PMC9755513

[41] Aicher HD Wolff M Herwig U . Psychedelic therapy – refining the claim of a paradigm shift. Int Rev Psychiatry. (2024) 36:1–8. doi: 10.1080/09540261.2024.2410853, PMID: 39980220

[42] Sumner R Lukasiewicz K . Psychedelics and neural plasticity. BMC Neurosci. (2023) 24:35. doi: 10.1186/s12868-023-00809-0, PMID: 37391744 PMC10311817

[43] Lepow L Morishita H Yehuda R . Critical period plasticity as a framework for psychedelic-assisted psychotherapy. Front Neurosci. (2021) 15:710004. doi: 10.3389/fnins.2021.710004, PMID: 34616272 PMC8488335

[44] Hultgren J Hafsteinsson MH Brulin JG . A dose of therapy with psilocybin - A meta-analysis of the relationship between the amount of therapy hours and treatment outcomes in psychedelic-assisted therapy. Gen Hosp Psychiatry. (2025) 96:234–43. doi: 10.1016/j.genhosppsych.2025.07.020, PMID: 40782561

[45] Schlag AK Aday JS Salam I Neill JC Nutt DJ . Adverse effects of psychedelics: From anecdotes and misinformation to systematic science. J Psychopharmacol (Oxford England). (2022) 36:258–72. doi: 10.1177/02698811211069100, PMID: 35107059 PMC8905125

[46] Griffiths RR Johnson MW Carducci MA Umbricht A Richards WA Richards BD . Psilocybin produces substantial and sustained decreases in depression and anxiety in patients with life-threatening cancer: A randomized double-blind trial. J Psychopharmacol (Oxford England). (2016) 30:1181–97. doi: 10.1177/0269881116675513, PMID: 27909165 PMC5367557

[47] Armstrong SB Levin AW Xin Y Horan JC Luoma JB Nagib P . Differences in attitudes and beliefs about psychedelic-assisted therapy among social workers, psychiatrists, and psychologists in the United States. J Psychedelic Stud. (2023) 7:61–7. doi: 10.1556/2054.2023.00245, PMID: 29951292

[48] Hartogsohn I . Modalities of the psychedelic experience: Microclimates of set and setting in hallucinogen research and culture. Transcultural Psychiatry. (2022) 59:579–91. doi: 10.1177/13634615221100385, PMID: 35818775

[49] Langlitz N Dyck E Scheidegger M Repantis D . Moral psychopharmacology needs moral inquiry: the case of psychedelics. Front Psychiatry. (2021) 12:680064. doi: 10.3389/fpsyt.2021.680064, PMID: 34408677 PMC8365088

[50] Olson DE . The subjective effects of psychedelics may not be necessary for their enduring therapeutic effects. ACS Pharmacol Trans Sci. (2021) 4:563–7. doi: 10.1021/acsptsci.0c00192, PMID: 33861218 PMC8033607

[51] Palitsky R Kaplan DM Peacock C Zarrabi AJ Maples-Keller JL Grant GH . Importance of integrating spiritual, existential, religious, and theological components in psychedelic-assisted therapies. JAMA Psychiatry. (2023) 80:743–9. doi: 10.1001/jamapsychiatry.2023.1554, PMID: 37256584

[52] Caporuscio C Kind A . “ Therapist, trip sitter or guide? A second-person perspective on psychedelic-assisted psychotherapy.” In: Lovering R , editor. The palgrave handbook of philosophy and psychoactive drug use. Palgrave Macmillan, Cham: Springer Nature Switzerland (2024). p. 513–30. doi: 10.1007/978-3-031-65790-0_25, PMID:

[53] Smith CL Sackett N Stark BC Dinh V Romesburg EW Roll J . Understanding psychedelic-assisted psychotherapy providers’ Perspective and insights: A qualitative analysis. Psychedelic Med (New Rochelle N.Y.). (2024) 2:153–60. doi: 10.1089/psymed.2023.0074, PMID: 40051684 PMC11658670

[54] Cavarra M Falzone A Ramaekers JG Kuypers KPC Mento C . Psychedelic-assisted psychotherapy-A systematic review of associated psychological interventions. Front Psychol. (2022) 13:887255. doi: 10.3389/fpsyg.2022.887255, PMID: 35756295 PMC9226617

[55] Kittur ME Burgos M LA Jones BDM Blumberger DM Mulsant BH Rosenblat JD . Mapping psilocybin therapy: A systematic review of therapeutic frameworks, adaptations, and standardization across contemporary clinical trials. J Affect Disord. (2025) 391:119952. doi: 10.1016/j.jad.2025.119952, PMID: 40684956

[56] Guss J Krause R Sloshower J . The yale manual for psilocybin-assisted therapy of depression (using acceptance and commitment therapy as a therapeutic frame) (2020). Available online at: https://osf.io/preprints/psyarxiv/u6v9y_v1.

[57] Yaden DB Earp D Graziosi M Friedman-Wheeler D Luoma JB Johnson MW . Psychedelics and psychotherapy: cognitive-behavioral approaches as default. Front Psychol. (2022) 13:873279. doi: 10.3389/fpsyg.2022.873279, PMID: 35677124 PMC9169963

[58] Fischman LG . Seeing without self: Discovering new meaning with psychedelic-assisted psychotherapy. Neuropsychoanalysis. (2019) 21:53–78. doi: 10.1080/15294145.2019.1689528, PMID: 41891072

[59] Watts R Luoma JB . The use of the psychological flexibility model to support psychedelic assisted therapy. J Contextual Behav Sci. (2020) 15:92–102. doi: 10.1016/j.jcbs.2019.12.004, PMID: 41895306

[60] McGovern HT Grimmer HJ Doss MK Hutchinson BT Timmermann C Lyon A . An Integrated theory of false insights and beliefs under psychedelics. Commun Psychol. (2024) 2:69. doi: 10.1038/s44271-024-00120-6, PMID: 39242747 PMC11332244

[61] Bøhling F . Psychedelic pleasures: An affective understanding of the joys of tripping. Int J Drug Policy. (2017) 49:133–43. doi: 10.1016/j.drugpo.2017.07.017, PMID: 28918193

[62] Gandy S Forstmann M Carhart-Harris RL Timmermann C Luke D Watts R . The potential synergistic effects between psychedelic administration and nature contact for the improvement of mental health. Health Psychol Open. (2020) 7:2055102920978123. doi: 10.1177/2055102920978123, PMID: 33335742 PMC7724423

[63] O’Callaghan C Hubik DJ Dwyer J Williams M Ross M . Experience of music used with psychedelic therapy: A rapid review and implications. J Music Ther. (2020) 57:282–314. doi: 10.1093/jmt/thaa006, PMID: 32227084

[64] Aicher HD Röskamp A Moser M Brand M . “ The Role and Ethics of Touch and Non-touch in Psychedelic-Assisted Therapy.” In: Current Topics in Behavioral Neurosciences. Berlin, Heidelberg: Springer. (2025). pp. 1–36. doi: 10.1007/7854_2025_608, PMID: 41272341

[65] Back A Myers S Guy J Perez J Lazar Thorn L McGregor B . Evolving guidelines for the use of touch during a clinical trial of group psilocybin-assisted therapy. Psychedelic Med. (2024) 2:psymed.2023.0069. doi: 10.1089/psymed.2023.0069, PMID: 40051480 PMC11658378

[66] Phelan JE . Exploring the use of touch in the psychotherapeutic setting: A phenomenological review. Psychother (Chicago Ill.). (2009) 46:97–111. doi: 10.1037/a0014751, PMID: 22122573

[67] Woolfe S . The Ethics of Touch in Psychedelic Therapy: Explore ethical considerations of therapeutic touch in psychedelic therapy. Learn how it aids healing and the importance of clear boundaries (2024). Available online at: https://psychedelic.support/resources/ethics-therapeutic-touch-in-psychedelic-therapy/ (Accessed November 6, 2024).

[68] Smith WR Sisti D . Ethics and ego dissolution: The case of psilocybin. J Med Ethics. (2020) 47:807–14. doi: 10.1136/medethics-2020-106070, PMID: 32461241 PMC9202314

[69] Hall W . Ending the silence around psychedelic therapy abuse. Cambridge, MA, USA: madinamerica.com (2021). Available online at: https://www.madinamerica.com/2021/09/ending-silence-psychedelic-therapy-abuse/ (Accessed November 7, 2024).

[70] Multidisciplinary Association for Psychedelic Studies . Participant experiences & “Cover story” (2022). Available online at: https://maps.org/2022/03/01/participant-experiences-cover-story/ (Accessed November 6, 2024).

[71] Luoma JB Allen LR Gold V Stauffer CS . Getting in touch with touch: the importance of studying touch in MDMA-assisted therapy and the development of a new self-report measure. Psychedelic Med. (2024) 2:25–32. doi: 10.1089/psymed.2023.0033, PMID: 40051757 PMC11658647

[72] Baier AL Kline AC Feeny NC . Therapeutic alliance as a mediator of change: A systematic review and evaluation of research. Clin Psychol Rev. (2020) 82:101921. doi: 10.1016/j.cpr.2020.101921, PMID: 33069096

[73] Avasthi A Grover S Nischal A . Ethical and legal issues in psychotherapy. Indian J Psychiatry. (2022) 64:S47–61. doi: 10.4103/Indianjpsychiatry.Indianjpsychiatry_50_21, PMID: 35599651 PMC9122134

[74] Wolff M Rutrecht H Mertens LJ Meijer AA Beck J Pérez S . Common factors in altered states: Understanding psychedelic therapy through the lens of Grawe’s general change mechanisms. psychol Rev. (2025) 132:1467–92. doi: 10.1037/rev0000589, PMID: 41066253

[75] Phelps J . Developing guidelines and competencies for the training of psychedelic therapists. J Humanistic Psychol. (2017) 57:450–87. doi: 10.1177/0022167817711304, PMID: 41883860

[76] Eisner BG . Set, setting, and matrix. J Psychoactive Drugs. (1997) 29:213–6. doi: 10.1080/02791072.1997.10400190, PMID: 9250949

[77] Hartogsohn I . Set and setting, psychedelics and the placebo response: An extra-pharmacological perspective on psychopharmacology. J Psychopharmacol (Oxford England). (2016) 30:1259–67. doi: 10.1177/0269881116677852, PMID: 27852960

