# Supplementary material for: Synergies in psychedelic-assisted therapy: a qualitative interview study of psychotherapeutic processes
Source: Front Psychiatry. 2026 Apr 1;17:1771726. doi: 10.3389/fpsyt.2026.1771726 (PMC13079574; doi:10.3389/fpsyt.2026.1771726)
Supplement: Supplementary file 1 [file Supplementaryfile1.zip › Interview Guide - English Translation.pdf]

Interview Guide (translated version):
Exploring the Role of Psychotherapy in Psychedelic-Assisted Therapy
Authors: Jonathan Stellmacher & Kae Eichel

Topic	Core Question	Checklist of Key Aspects	Probing Questions	Prompting and Redirecting Questions
Introduction	- Introduction<br>- Who I am<br>- Rationale of the research<br>- Aim of the study<br>- Confidentiality<br>- Data handling<br>- Duration<br>**Quant. data collection:**<br>- (Name)<br>- Age<br>- Gender<br>- Years of professional experience overall<br>- Years of professional experience in PAT<br>- Number of PAT patients treated<br>- Number of PAT sessions conducted<br>- Which substances do you work with in PAT?<br>- Do you work in individual or group settings?<br><br>What is your therapeutic background?<br><br>Imagine a "typical" PAT treatment. What would it look like?			
Therapeutic stance and preparation	**What therapeutic stance do you aim to adopt before entering a PAT treatment?**<br><br>**How do you shape the therapeutic setting in a PAT treatment?**	- therapeutic stance<br>- therapeutic relationship<br>- design of setting<br>…have been mentioned	What psychotherapeutic stance do you associate with PAT?<br><br>How do you see your role in relation to the patient you are treating?<br><br>How do you shape the therapeutic relationship in PAT treatments?	How do you prepare yourself for a PAT treatment?<br>What is the intention behind this?<br><br>What do you consider particularly important during a PAT treatment, and why?

Effect of stance and preparation	**What significance does your stance have for the PAT treatment?**	The connection between stance/setting and impact was described.	How does your therapeutic stance influence your work with patients?<br><br>How does the setting influence your work?<br><br>How do you perceive the relation between these aspects (stance, setting, relationship) and the course of treatment?	
Methods	**Do you use specific therapeutic methods during PAT treatment? Which methods do you apply?**	Therapeutic methods were described, or reasons were provided for why no specific methods are applied.<br>For all phases:<br>- Before<br>- During<br>- After	How do patients' life circumstances or expectations regarding psychedelic treatment influence the process in this context?	How do these methods differ from conventional psychotherapy processes without psychedelics?
Effect of methods	**To what extent are specific psychotherapeutic methods relevant for PAT treatment?**	The effects of methods were described, if such methods were applied.	How do these methods affect the treatment process?<br><br>Are there particular statements or conceptions that you pay special attention to in this context? If so, which ones and why?	Suppose you behaved in a friendly manner during PAT treatment, but without any specific reference to the psychedelic treatment. What effect do you think this would have?
General / additional questions	**How do individual and group settings differ in PAT?**<br><br>**How do psychedelics affect the therapeutic relationship?**			
Perspectives	**What do psychedelics as substances contribute to the therapeutic process?**	It was explored whether psychedelics are perceived as nonspecific amplifiers or as constituting a distinct class of psychotherapeutic experiences.	What is the difference between treatments conducted under the influence of psychedelics and treatments without psychedelics?	What effects do psychedelics have on patients?

	**To what extent, if at all, does psychotherapy influence the psychedelic experience?**	The perceived relevance of the neurobiological components of PAT was assessed.	What role does the neurobiological effect of psychedelics play in PAT?	Let us assume that it was possible to ensure that patients feel safe in relation to the psychedelic experience without psychotherapeutic support. How would the use of psychedelics affect them?
Comparison of Perspectives	**Which of the following statements do you agree with most? "Psychedelics primarily exert psychopharmacological effects", " Psychedelics amplify specific psychotherapeutic processes occurring in therapy." "PAT constitutes its own class of psychotherapeutic processes." How did you arrive at this conclusion?**			

Perspectives:

1. Psychedelic-Assisted Therapy constitutes its own class of psychotherapeutic processes.
2. Psychedelics are unspecific catalysts of specific psychotherapeutic processes.
3. Psychedelics work exclusively through psychopharmacology, psychotherapy is necessary to ensure the safety of this process.
