# Supplementary material for: Synergies in psychedelic-assisted therapy: a qualitative interview study of psychotherapeutic processes
Source: Front Psychiatry. 2026 Apr 1;17:1771726. doi: 10.3389/fpsyt.2026.1771726 (PMC13079574; doi:10.3389/fpsyt.2026.1771726)
Supplement: Supplementary file 1 [file Supplementaryfile1.zip › Interview Guide - German.PDF]

Interviewleitfaden: Exploring the Role of Psychotherapy in Psychedelic-Assisted Therapy
Autor*innen: Jonathan Stellmacher & Kae Eichel

Thema	Leitfrage	Check Aspekte	Konkretisierende Frage	Aufrechterhaltungs- und Steuerungsfragen
Einstieg	- Einleitung<br>- Wer ich bin<br>- Forschungsgrund<br>- Ziel der Studie<br>- Vertraulichkeit<br>- Umgang mit Daten<br>- Dauer<br>**Quant. Datenerhebung:**<br>- (Name)<br>- Alter<br>- Geschlecht<br>- Jahre Berufserfahrung allgemein<br>- Jahre Berufserfahrung PAT<br>- Anzahl PAT Pat.<br>- Anzahl PAT Stunden<br>- Mit welchen Substanzen arbeiten Sie innerhalb der PAT?<br>- Arbeiten Sie mit Gruppen/individuell?<br><br>Was ist Ihr therapeutischer Hintergrund?<br><br>Stellen Sie sich eine PAT Behandlung vor, die "typisch" abläuft. Wie würde die aussehen?			
PT Haltung und Vorbereitung	**Welche Haltung streben Sie an, bevor Sie in eine PAT Behandlung gehen?**<br><br>**Wie gestalten Sie das Setting bei einer PAT-Behandlung?**	- Psycho-therapeutische Haltung<br>- therapeutische Beziehung,<br>- Setting -gestaltung<br>…sind zur Sprache gekommen.	Welche psychotherapeutische Haltung verbinden Sie mit der PAT?<br><br>Wie sehen Sie Ihre Rolle in Bezug auf den*die Patient*in die sie behandeln?<br><br>Wie gestalten Sie die therapeutische Beziehung für eine PAT?	Wie bereiten Sie sich auf eine PAT Behandlung vor?<br>Auf was zielt das ab?<br><br>Was halten Sie bei der Durchführung einer PAT für besonders wichtig und warum?

Wirkung der Haltung und Vorbereitung	**Welche Bedeutung hat diese Einstellung für die PAT Behandlung?**	Zusammen-hang zwischen Wirkung und Rolle/ Setting wurde beschrieben.	Wie wirkt sich Ihre Haltung auf Ihre Arbeit mit den Patient*innen aus?<br><br>Wie wirkt sich das Setting darauf aus?<br><br>Wie sehen Sie das Verhältnis zwischen diesen Aspekten (Haltung, Setting, Beziehung) und dem Verlauf der Behandlung?	
Methoden	**Nutzen Sie spezifische Therapiemethoden im Verlauf der PAT-Behandlung?**<br>**Welche Methoden wenden Sie an?**	Erläuterung von Therapie-methoden ist erfolgt, bzw. es wurde erklärt, warum keine spezifischen Methoden angewendet werden.<br>Für alle Phasen:<br>- Vorher<br>- Während<br>- Danach	Wie wirken sich in diesem Kontext Lebensumstände oder Vorstellungen der Patient*innen in Bezug auf die psychedelische Behandlung aus?	Wie unterscheiden sich diese Methoden von üblichen Psychotherapieprozessen ohne Psychedelika?
Wirkung der Methoden	**Inwiefern sind spezifische psychotherapeutische Methoden für eine PAT Behandlung relevant?**	Auswirkungen von Methoden wurde beschrieben, falls Methoden angewendet werden.	Wie wirken sich diese auf den Behandlungsprozess aus?<br><br>Gibt es Aussagen oder Vorstellungen, denen Sie in diesem Kontext besondere Aufmerksamkeit schenken? Falls ja, welche und warum?	Angenommen Sie würden sich bei einer PAT Behandlung freundlich, aber ohne spezifischen Bezug auf die psychedelische Behandlung verhalten. Welche Auswirkungen hätte das Ihrer Meinung nach?
Allgemeine Fragen/ Extra-Fragen	**Wie unterscheiden sich Einzel-/Gruppensettings bei der PAT voneinander?**<br><br>**Wie wirken sich Psychedelika auf die therapeutische Beziehung aus?**			

Perspektiven	**Was tragen Psychedelika als Substanz zu dem Therapieprozess bei?**	Es wurde heraus-gearbeitet, ob Psychedelika unspezifischer Verstärker, oder als spezifische Klasse an PT Erfahrungen wahrgenommen wird.	Was ist der Unterschied zwischen Behandlungen die unter dem Einfluss von Psychedelika stattfinden im Vergleich zu Behandlungen ohne Psychedelika?	Welche Wirkung haben Psychedelika auf die Patient*innen?
	**Inwiefern hat die Psychotherapie einen Einfluss auf die Psychedelische Erfahrung?**	Die wahr-genommene Bedeutung der neuro-biologischen Komponenten der PAT wurde erfasst.	Welche Rolle spielt die neurobiologische Wirkung der Psychedelika bei der PAT?	Nehmen wir an, man könnte gewährleisten, dass Patient*innen sich im Bezug auf die psychedelische Erfahrung sicher fühlen, ohne psychotherapeutische Begleitung. Wie würde der Konsum von Psychedelika wirken?
Perspektiven-vergleich	**Welcher Aussage oder welchen Aussagen stimmen Sie am meisten zu: "Psychedelika wirken vor allem psychopharmakologisch", "Psychedelika verstärken in der PT stattfindende spezifische Prozesse" "PAT stellt eine eigene Form von psychotherapeutischen Prozessen dar." Wie kamen Sie zu dieser Antwort?**			

Perspektiven:

1. Psychedelika-assistierte Therapie stellt eine eigene Klasse an psychotherapeutischer Prozesse dar.
2. Psychedelika sind unspezifische Verstärker von spezifischen PT Prozessen.
3. Psychedelika wirken rein psychopharmakologisch, die PT ist wichtig um den Prozess sicher zu gestalten
