# Supplementary material for: Synergies in psychedelic-assisted therapy: a qualitative interview study of psychotherapeutic processes
Source: Front Psychiatry. 2026 Apr 1;17:1771726. doi: 10.3389/fpsyt.2026.1771726 (PMC13079574; doi:10.3389/fpsyt.2026.1771726)
Supplement: Supplementary file 1 [file Supplementaryfile1.zip › Additional Thematic Quotes.pdf]

<u>Additional Thematic Quotes</u>

Supplementary material for the article

Synergies in Psychedelic-Assisted Therapy:
A Qualitative Interview Study of Psychotherapeutic Processes

Authors

Jonathan Stellmacher, Christopher Schmidt, Helena Dorothea Aicher, Kae Eichel, Eva-Lotta Brakemeier, Uwe Herwig

Interviews conducted by

Jonathan Stellmacher

Note. The identifiers preceding each quote (e.g., "D1") correspond to statements in the main article that reflect the content illustrated by the quotes presented here.

D1. *The attitude of the patients should be one of, how shall I put it, responsible curiosity. […] And the willingness to understand what happens as part of themselves.* (Therapist 6)

D2. *[Before the dosing session we] revisit these goals, go over the mantra or the recommendations for the day again. Such as letting go, trusting, and being curious.* (Therapist 7)

D3. *Nausea is often a result of resistance, of not being able or willing to engage, and so on. On a cognitive level, there's the fear.* (Therapist 2)

D4. *I really find it difficult how things are today with people reaching out for PAT. Having already made the decision themselves that they want it.* (Therapist 3)

D5. *After the second time, she said: "I want psilocybin." I told her to stay focused and that we would work on these topics, and then after five or six MDMA sessions, we switched to psilocybin. I kept telling her that nothing would change; it's a different experience, but it won't be a "Hallelujah" moment. And she always replied, "Yes, yes." But when she took it, she was in tears for six hours.* (Therapist 2)

D6. *Sometimes I feel that many people have more empowering experiences at the beginning, which are somewhat positive. And then, as you continue, more challenging experiences emerge. The initial experiences act as a kind of foundation to gather strength, and later, it's about peeling back layers.*

But there are also people who experience a lot of frustration, and at some point, something starts to shift—perhaps because their […] expectations are no longer as strong. (Therapist 1)

M1. *We do breathing exercises or body scan exercises. […] It simply means turning your attention to your body. You can always re-anchor yourself in the body.* (Therapist 6)

M2. *Letting thoughts pass like clouds, letting them come and go or practicing "letting go". Simply observing things with attention and curiosity. Even when fear arises. Just notice what's there without judging. And if a judgment comes, welcome it too and let it pass.* (Therapist 1)

M3. *It's like a way to normalize feelings of anxiety and nervousness. That, of course, plays a role too. The goal is to make the substance experience as comfortable as possible.* (Therapist 7)

O1. *Well, I'd say a nice room, a pleasant atmosphere, and above all, a space where I feel comfortable. […] We have a shared practice room that my colleague and I arrange in the morning. There's tea, a bouquet of flowers, and a candle for those who like that. We also make sure it's not too noisy outside, if possible. In our case, we're fortunate to be close to a green area where you can step outside without having to drive across the city. When that's possible, I find it very convenient because sometimes it's just nice to get some fresh air. Additionally, we pay attention to the music, making sure the speakers are set up well. These are all the little details, like ensuring there's enough water available.* (Therapist 5)

O2. *I think the setting is about predictability—what comes next, clarity, and a kind of basic control, so that one can somewhat engage in the experience.* (Therapist 4)

O3. *[Being] not very directive; rather, it is more supportive and primarily focused on maintaining presence. It's about being truly there, open to whatever comes.* (Therapist 5)

O4. *[…] staying with oneself, parking the body, not dissociating, being aware of feelings without necessarily acting them out. […]* (Therapist 2)

G1. *[It is important to be] able to regulate oneself in close contact with others, so as not to feel overwhelmed suddenly and pull away abruptly. Or to think: 'Oh, what do I have to do now? Oh dear, I have to comfort this person?' It's about having the ability to connect and then tune into the contact.* (Therapist 3)

G2. *Like when someone regresses and becomes like a four-year-old child, for example, sometimes you just need to hold them.* (Therapist 2)

G3. *Sounds really create this trance-like state. Trance is, in a way, an opening, an expansion. It has this sense of spaciousness.* (Therapist 3)

G4. *When I say "in silence", I mean "not a lot of conversation". But music: I have a pre-made playlist that I play, but I also adjust it as needed.* (Therapist 7)

G5. *In the garden, I had picked up two rosemary plants. […] At some point during the journey, I gave it to him to smell. […] What I didn't know at all was that he had worked professionally with farmers in Morocco who grew rosemary. […] A lot came out of that later.* (Therapist 3)

G6. *And no decision-based questions. […] "Would you prefer orange juice or water?" "Wait, what is water again? And what is orange juice? And where on earth am I supposed to come up with a decision?" (laughter)* (Therapist 3)

C1. *And then later in the early afternoon, there are also cognitive discussions where the content is addressed.* (Therapist 7)

C2. *When you have eight people who have the time to share their process and what they experienced and how they perceived different aspects, all that what a group enables - that is often really a great gift.* (Therapist 3)

C3. *We also encourage the patients to write a protocol about what they experienced.* […] *it's about helping the patients to structure these very complex and challenging, exhausting, and multifaceted experiences they have during the substance experience. […] I believe that alone is already a therapeutic process—kind of like capturing the indescribable to some extent.* (Therapist 7)

E1. *Someone had already noticed that it was really important for them at work to have a bit more privacy in an open-plan office. And while they had known this for a long time, they had never really taken it seriously. Then the concrete question was: how can they actually make that happen? […] They were able to make that arrangement, and it significantly reduced the stress of working eight hours a day.* (Therapist 5)

E2. *And when the patients bring certain resources to be able to work with the experience, I do think that the substance experience itself has potential. But I think, for many patients, that's not enough.* (Therapist 1)

E3. *Let's assume he had stayed in those fantasies and hadn't had the opportunity to discuss it with me; then it would have been like an unfinished gestalt all the time.* (Therapist 5)

E4. *[When] I suddenly become, I don't know, the evil mother during the experience or something, that this may be the case in the moment, but it can also be integrated and discussed afterward.* (Therapist 1)

E5. *Or an experience of: "I feel like I'm going crazy or I will never come down from this." And somehow the fear of losing control. This can later be processed to understand what this fear of losing control really is.* (Therapist 1)

E6. *An intervention might be perceived as intrusive or disruptive, or there might be something needed that doesn't come. And then […] you can talk about it. What was needed, what was bothersome? This is part of the therapy, that one talks about how something was experienced. This is essential because it reflects much that cannot yet be understood.* (Therapist 4)

P1. *[In PAT there is much more] intensity and density of experiences. […] Whereas in a classic therapy without substances, the defense mechanisms are simply more active, resulting in less intensity, less information, and less... You might only get a hint that something is wrong or that something might not be understood with what we've just discussed. [It] doesn't fit together. Then you can follow up on that. With the substance, this often becomes glaringly obvious.* (Therapist 4)

P2. *Psychedelics contribute to a state, in which many things can no longer be avoided in the therapy process.* (Therapist 7)

P3. *With one patient a really deep process started very quickly. I wasn't quite sure where she was, but she really began to thrash around, and there was a real danger that she could hurt herself.* (Therapist 5)

T1. *So there must fundamentally be a relationship of trust. And when a changed state of consciousness occurs, and they suddenly see me as a gorilla or who knows what, they need to know in the back of their mind that this is the therapist, he is okay, he means me no harm, etc.* (Therapist 2)

T2. *I also need to be able to trust the patients, that they are capable […] to establish a trusting relationship with me. [...] In this state, they may need to be willing to listen to me and not do something if I say it's dangerous, or if it might be self-destructive, or if an aggressive behaviour disturbs others.* (Therapist 6)

T3. *There is the initial preparation phase, where you get to know the patients, take their medical history, and explore conflicts. […] During this phase, a foundation of trust also needs to be established.* (Therapist 7)

T4. *Each time before a PAT, I also tell the patients a little bit about myself. […] Just some superficial personal things, so that they have a bit of a sense of who I am. And I believe that's valuable for building trust.* (Therapist 7)

T5. *I usually start with MDMA. It has this empathogenic effect and also this anxiety-reducing effect, which can further deepen the therapeutic relationship. It also enhances trust because the patients expose themselves to me to a certain degree.* (Therapist 2)

T6. *I also find it interesting that it becomes even more personal, as people naturally perceive a lot from me […] [They] ask things like, 'Are you sad?' This means they ask questions that a patient would rarely ask otherwise. (*Therapist 3)

X1. *You can never know where the journey will lead, really. It may be that it involves exploration. It could also be about the specific topic you set out to address. However, something entirely different might come up, including mystical experiences that are beyond words. Anything is possible, from various emotional states—pleasant or unpleasant—to physical reactions.* (Therapist 5)

X2. *[He suddenly realized] 'Oh my God. My father was only 25, and now I'm 35. Oh damn, he was so young when he became a father. Damn, I wouldn't have wanted that either'.* (Therapist 3)

X3. *Last time, she also had a feeling of inner warmth and security. It was the first time she experienced a sense of home or safety, which she hadn't felt in her childhood, where she was only confronted with a punitive and rigid environment.* (Therapist 2)

X4. *'As a child, this and that happened. But it's really not that bad'; that's the adult perspective. Then, in a psychedelic session, a regressive experience occurs where the person feels like a 3-year-old and truly senses how terrible it was.* (Therapist 1)

X5. *Assuming that traumas are well protected in the psyche, there is the idea and experience that psychedelics somewhat loosen this protection. Because access to what has been experienced or lived is possible with less fear or is more resourceful. And that increases the intensity that a patient can endure.* (Therapist 4)

X6. *They are blocked on the mat for a day, having just experienced fear all day or trying to fight against the fear.* (Therapist 6)

A1. *I think often not much will change on its own afterward if there's no ongoing psychotherapy. […] However, the studies show very different results—like one or two peak experiences, followed by one or two integration sessions, and then healed. Personally, I've rarely seen that happen.* (Therapist 5)

A2. *They say that this psilocybin or LSD experience was something they've never encountered before, and it's unique. And while uniqueness alone doesn't inherently have therapeutic value, I believe that the intensity of such an experience, precisely because it's so unique, provides the opportunity to engage deeply with it. That's where the therapeutic potential lies.* (Therapist 6)

A3. *They can, of course, have a purely psychopharmacological effect in certain situations.* (Therapist 1)

A4. *If we assume that traumas are relatively protected within the psyche, the idea and experience is that psychedelics somewhat loosen this protection. This allows for a more anxiety-free or resource-rich access to experiences or past encounters.* (Therapist 4)

A5. *It's not necessarily the case that I would say it must always be connected with psychotherapy. However, for the people I treat in psychiatric practice, it definitely is.* (Therapist 4)

A6. *But I mean, we all know that these are all people who have severe mental illnesses, and they need a bit more support.* (Therapist 3)

A7. *I would say that the substance likely does have pharmacological effects, allowing for synapses to form and connections to open up. However, in my view, it is truly a combination of psychotherapy and pharmacological effect.* (Therapist 5)

A8. *The increase in neuroplasticity and the tendency for patients to experience instability or short-term lability when using these substances underscores the importance of conducting this within the context of psychotherapy. […] Therefore, instead of reinforcing negative behavioural patterns—which could happen without psychotherapy—it makes sense to embed this experience within a therapeutic framework.* (Therapist 7)
